# Effects of moderate-to-vigorous physical activity on the associations between an insulin resistance surrogate and incident cardiovascular disease and all-cause mortality: a UK Biobank cohort study

**DOI:** 10.3389/fendo.2025.1709135

**Published:** 2025-11-05

**Authors:** Ying Zhu, Tianci Yao, Hongyu Yan, Qinmei Ke

**Affiliations:** ^1^ Department of Geriatrics, Union Hospital, Tongji Medical College, Huazhong University of Science and Technology, Wuhan, China; ^2^ Department of Endocrinology, Yueyang Central Hospital, Yueyang, China

**Keywords:** moderate to vigorous physical activity, insulin resistance, triglyceride glucose-waist height ratio index, cardiovascular disease, all-cause mortality

## Abstract

**Background:**

Moderate-to-vigorous physical activity (MVPA) and insulin resistance (IR) are associated with cardiovascular disease (CVD). It remains unclear whether different durations of MVPA can modify the associations of the triglyceride glucose-waist height ratio (TyG-WHtR) index, a surrogate for IR, with incident CVD and all-cause mortality, and whether MVPA levels beyond guideline recommendations provide additional benefit.

**Methods:**

This cohort study included 299,928 adults from the UK Biobank study who were free of prevalent CVD at baseline and had complete data on MVPA, the TyG-WHtR index, and relevant covariates. The Cox proportional hazards model was used to assess the independent and joint associations of MVPA and TyG-WHtR with incident CVD and all-cause mortality. A product term of MVPA (< 150, 150–299, 300–599, and ≥ 600 min/week) and TyG-WHtR (tertiles) was included in the model to assess multiplicative interaction.

**Results:**

During a median follow-up of 13.8 and 13.6 years, 27,342 CVD cases and 21,258 deaths were observed. MVPA demonstrated a reverse J-shaped association with incident CVD, with a cutoff point at 261.71 min/week, whereas an L-shaped association was observed for all-cause mortality, with risk reduction plateauing at 217.00 min/week. Elevated TyG-WHtR was positively associated with increased risks of incident CVD and all-cause mortality. No significant interaction was found for incident CVD, whereas an interaction effect was observed between 150–299 min/week MVPA and TyG-WHtR tertile 2 on all-cause mortality (HR for interaction, 0.89; 95% CI, 0.81–0.97; *p* for interaction = 0.012). Compared with the reference group (< 150 min/week MVPA and TyG-WHtR tertile 3), all other combined groups were associated with lower risks. Among participants with MVPA levels of 150–299, 300–599, and ≥ 600 min/week, the extent of risk reduction for these outcomes was similar.

**Conclusions:**

Our findings highlight the importance of engaging in guideline-recommended MVPA (150–299 min/week) to reduce all-cause mortality risk, particularly among individuals with moderate IR (TyG-WHtR, 7.01–8.03), who are more likely to benefit. Furthermore, the protective effects of higher levels of MVPA against IR-related risks were consistent with those of guideline-recommended MVPA.

## Introduction

Cardiovascular disease (CVD) remains one of the leading global causes of death and healthcare costs, imposing a substantial burden on healthcare systems worldwide (1, 2). Of particular concern is the continuous increase in the global burden of CVD, driven largely by unhealthy lifestyles, including lack of physical activity (PA), and metabolic risk factors such as dyslipidemia, glucose intolerance, and obesity (1–4).

Glycolipid metabolic disorders and obesity are strongly associated with insulin resistance (IR) (5–8), which is an important risk factor for CVD and mortality (4, 9, 10). Although the hyperinsulinemic–euglycemic clamp test is regarded as the gold standard for IR, it is not feasible for large-scale studies due to its complexity and invasiveness (11). The homeostasis model assessment of IR (HOMA-IR), a common surrogate, has limited application in individuals receiving insulin treatment or in those without functioning beta cells (12). Given these limitations, the triglyceride–glucose (TyG) index has emerged as a simple, convenient, and cost-effective surrogate for IR by integrating triglyceride (TG) and fasting blood glucose (FBG) (13). Furthermore, the triglyceride glucose-waist height ratio (TyG-WHtR), which combines the TyG index and waist-to-height ratio (WHtR), has been shown to perform better than the TyG index alone in reflecting IR and related dysmetabolism (14–17), as it integrates information on both glycolipid metabolism and central adiposity (18, 19). For CVD and mortality risk, studies have also shown that TyG-WHtR is a superior predictor compared with other commonly used surrogates, such as HOMA-IR and other TyG-related indices (15, 16, 20–26).

Evidence indicates that PA plays an important role in mitigating metabolic syndrome, including obesity and IR (27). Our previous study found that at least 150 min/week of moderate-to-vigorous physical activity (MVPA), as recommended by guidelines (28, 29), effectively mitigated the risks of all-cause mortality and myocardial infarction associated with IR, regardless of sedentary behavior (30). However, it remains unclear whether varying durations of MVPA can modify the associations of IR with incident CVD and all-cause mortality, and whether MVPA levels beyond guideline recommendations (300–599 and ≥ 600 min/week) could further reduce these IR-related risks.

Therefore, this study aimed to investigate the independent and joint associations of MVPA and TyG-WHtR with the risks of incident CVD and all-cause mortality, and to assess potential interactions between MVPA and TyG-WHtR among participants from the UK Biobank.

## Materials and methods

### Study design and participants

UK Biobank is a population-based prospective cohort study that recruited 502,356 participants aged 37 to 73 years between 2007 and 2010 from 22 assessment centers in England, Wales, and Scotland (31). Participants completed a touchscreen questionnaire, underwent physical measurements, and provided biological samples, as described in detail elsewhere (32, 33). UK Biobank was approved by the National Information Governance Board for Health and Social Care and the National Health Service North West Multi-Centre Research Ethical Committee (REF: 11/NW/03820), and all participants provided written informed consent before the baseline assessment (31).

Among the 502,356 participants, we excluded those with missing data on TG (*n* = 33,279), FBG (*n* = 72,913), WHtR (*n* = 2,268), MVPA (*n* = 94,227), or any covariates (*n* = 27,078), as well as those with prevalent CVD (including angina pectoris, myocardial infarction, heart failure, and stroke) at baseline. Additionally, extreme values at the 0.5% quantiles at both ends for MVPA were excluded. Ultimately, 299,928 participants were included in this study (**Supplementary Figure S1**). The diagnosis of prevalent CVD was obtained through self-reported disease history and linked hospital admissions data (International Statistical Classification of Diseases (ICD)-9: 410–414, 428, 430–434, 436; ICD-10: I20–I25, I50, I60–I64).

### Outcome variable

Incident CVD and all-cause mortality within the UK Biobank were defined using hospital admissions data and death registry data. The diagnosis of incident CVD was based on ICD-10 codes (I20–I25, I50, I60–I64). Hospital registry-based follow-up ended on 31 October 2022 in England, 31 August 2022 in Scotland, and 31 May 2022 in Wales. Dates of death were obtained from the death registry data up to 31 December 2022. Follow-up time for each participant was calculated from the date of baseline investigation until the date of CVD diagnosis, death, loss to follow-up, or end of the follow-up period, whichever occurred first.

### Exposure variable and covariates

MVPA was calculated by considering both the frequency and duration of weekly activities, as assessed through the touchscreen questionnaire at baseline. According to current PA guidelines, MVPA levels were classified as < 150, 150–299, 300–599, and ≥ 600 min/week (29).

The TyG-WHtR index was calculated as ln [TG (mg/dl) × FBG (mg/dl)/2] × WHtR, where WHtR was calculated as WC (cm)/standing height (cm). Peripheral venous blood samples were collected from all participants at baseline, and the collection procedures of the UK Biobank study were validated (32). TG and FBG concentrations were measured using clinical chemistry (Beckman Coulter AU5800). The coefficient of variation was less than 3% for TG and less than 2% for FBG.

Other covariates included age, sex, race, educational level, Townsend deprivation index, smoking status, drinking status, parental history of CVD, and self-reported use of antihypertensive drugs, lipid-lowering drugs, or insulin. Education level was classified as high (college or university degree) and low (including A levels/AS levels or equivalent, O levels/GCSEs or equivalent, CSEs or equivalent, NVQ or HND or HNC, or equivalent, and other professional qualifications). The Townsend deprivation index was computed using the most recent national census data corresponding to participants’ postcodes, with negative values indicating higher socioeconomic status (34). Smoking status was classified as current smoker or nonsmoker. Drinking status was classified as moderate drinking or other. Moderate drinking was defined as consuming ≤ 8 g/day for women and ≤ 16 g/day for men, based on the dietary guidelines in the UK (35).

### Statistical analysis

Incident CVD and all-cause mortality were recorded, and their incidence rates per 1,000 person-years were calculated based on the number of events and total follow-up time. The Cox proportional hazards model was used to estimate the hazard ratios (HRs) and 95% confidence intervals (CIs) for the outcomes. Proportional hazards assumptions were tested using a likelihood ratio test comparing models with and without time-dependent exposure, and no significant deviation from the assumption was observed. Linear trends were tested by assigning the median value of each group as a continuous variable. Additionally, a restricted cubic spline (RCS) regression model was used to examine the dose–response associations of MVPA levels and the TyG-WHtR index with the outcomes.

Furthermore, we conducted a stratified analysis by MVPA groups to explore the associations of the TyG-WHtR index with incident CVD and all-cause mortality among participants with different MVPA levels. A product term of MVPA (four groups) and TyG-WHtR index (tertiles) was additionally included in the multivariable-adjusted model to assess the multiplicative interaction. The HR (95% CI) of the product term was used as the measure of interaction on the multiplicative scale. To assess the joint associations, participants were conjointly reclassified into 12 groups based on MVPA (four groups) and the TyG-WHtR index (tertiles).

To test the robustness and potential variations in various subgroups, we repeated interaction and joint analyses stratified by age (< 65 and ≥ 65 years), sex (men and women), race (White and non-White), and education level (high and low).

Several sensitivity analyses were conducted. First, participants who experienced outcomes within the first 2 years of follow-up were excluded to reduce potential reverse causation. Second, multiple imputation was used to account for all missing covariates and assess the influence of missing data. Third, because sedentary behavior is strongly associated with PA, we further adjusted for sedentary behavior (< 6 and ≥ 6 h/day) in the multivariable-adjusted model. Finally, all analyses were repeated using the TyG-WC index, calculated as ln [TG (mg/dl) × FBG (mg/dl)/2] × WC (cm), instead of the TyG-WHtR index.

All analyses were performed using R statistical software, version 4.4.2. Two-sided tests with *p* < 0.05 were considered statistically significant.

## Results

### Baseline characteristics

Baseline characteristics of participants according to MVPA groups are shown in **Table 1**. Among the 299,928 participants, 162,735 (54.3%) were women, the mean (SD) age was 55.9 (8.0) years, the mean (SD) BMI was 27.2 (4.6) kg/m^2^, and the mean (SD) TyG-WHtR index was 7.6 (1.1). Participants with more MVPA were more likely to be older, men, White, leaner, nonsmokers, nondrinkers, and to have a lower TyG-WHtR index, no parental history of CVD, and not use drugs (antihypertensive drugs, lipid-lowering drugs, and insulin). Baseline characteristics of participants according to the tertiles of the TyG-WHtR index are displayed in **Supplementary Table S1**. Participants with a higher TyG-WHtR index were more likely to have less MVPA. The baseline characteristics of participants excluded and included in the analysis are displayed in **Supplementary Table S2**. The participants excluded were more likely to be older, women, more educated, more deprived, and to have more MVPA and a higher TyG-WHtR index.

**Table 1:** Baseline characteristics of the participants according to MVPA groups.

Variables	< 150 min/week (*n* = 116,280)	150–299 min/week (*n* = 51,815)	300–599 min/week (*n* = 61,512)	≥ 600 min/week (*n* = 70,321)	Total (*n* = 299,928)	*P-*value
Age (years)	55.7 (7.8)	55.7 (8.1)	55.8 (8.2)	56.6 (8.2)	55.9 (8.0)	< 0.001
Sex (%)						
Male	51,683 (44.4)	22,543 (43.5)	27,949 (45.4)	35,018 (49.8)	137,193(45.7)	< 0.001
Female	64,597 (55.6)	29,272 (56.5)	33,563 (54.6)	35,303 (50.2)	162,735 (54.3)	
Race (%)						
White	110,873 (95.4)	49,525 (95.6)	58,976 (95.9)	67,540 (96.0)	286,914 (95.7)	< 0.001
Black	1,410 (1.2)	677 (1.3)	759 (1.2)	862 (1.2)	3,708 (1.2)	
Asian	2,445 (2.1)	954 (1.8)	1,032 (1.7)	1,092 (1.6)	5,523 (1.8)	
Other	1,552 (1.3)	659 (1.3)	745 (1.2)	827 (1.2)	3,783 (1.3)	
Educational level (%)						
High	55,460 (47.7)	22,827 (44.1)	27,912 (45.4)	38,109 (54.2)	144,308 (48.1)	< 0.001
Low	60,820 (52.3)	28,988 (55.9)	33,600 (54.6)	32,212 (45.8)	155,620 (51.9)	
Townsend deprivation index (%)						
Most deprived	21,169 (18.2)	8,510 (16.4)	9,972 (16.2)	12,156 (17.3)	51,807 (17.3)	< 0.001
Intermediate deprived	70,561 (60.7)	31,703 (61.2)	37,745 (61.4)	43,264 (61.5)	183,273 (61.1)	
Least deprived	24,550 (21.1)	11,602 (22.4)	13,795 (22.4)	14,901 (21.2)	64,848 (21.6)	
BMI (kg/m^2^)	27.8 (5.1)	26.9 (4.5)	26.7 (4.3)	26.6 (4.2)	27.2 (4.6)	< 0.001
WHtR	0.9 (0.1)	0.9 (0.1)	0.9 (0.1)	0.9 (0.1)	0.9 (0.1)	< 0.001
Current smoking (%)						
No	103,810 (89.3)	47,425 (91.5)	56,446 (91.8)	63,524 (90.3)	271,205 (90.4)	< 0.001
Yes	12,470 (10.7)	4,390 (8.5)	5,066 (8.2)	6,797 (9.7)	28,723 (9.6)	
Moderate drinking (%)						
No	65,689 (56.5)	31,203 (60.2)	37,461 (60.9)	40,660 (57.8)	175,013 (58.4)	< 0.001
Yes	50,591 (43.5)	20,612 (39.8)	24,051 (39.1)	29,661 (42.2)	124,915 (41.6)	
MVPA (min/week)	47.3 (46.0)	212.2 (41.2)	416.2 (82.7)	1,234.9 (707.6)	429.89 (580.4)	< 0.001
Parental history of CVD (%)						
No	49,356 (42.4)	22,443 (43.3)	26,942 (43.8)	30,493 (43.4)	129,234 (43.1)	< 0.001
Yes	66,924 (57.6)	29,372 (56.7)	34,570 (56.2)	39,828 (56.6)	170,694 (56.9)	
Self-reported use of antihypertensive drugs (%)						
No	94,372 (81.2)	43,311 (83.6)	52,047 (84.6)	59,234 (84.2)	248,964 (83.0)	< 0.001
Yes	21,908 (18.8)	8,504 (16.4)	9,465 (15.4)	11,087 (15.8)	50,964 (17.0)	
Self-reported use of lipid-lowering drugs (%)						
No	99,683 (85.7)	45,368 (87.6)	54,315 (88.3)	61,972 (88.1)	261,338 (87.1)	< 0.001
Yes	16,597 (14.3)	6,447 (12.4)	7,197 (11.7)	8,349 (11.9)	38,590 (12.9)	
Self-reported use of insulin (%)						
No	115,037 (98.9)	51,463 (99.3)	61,116 (99.4)	69,825 (99.3)	297,441 (99.2)	< 0.001
Yes	1,243 (1.1)	352 (0.7)	396 (0.6)	496 (0.7)	2,487 (0.8)	
TG (mmol/L)	1.8 (1.1)	1.7 (1.0)	1.7 (1.0)	1.6 (1.0)	1.7 (1.0)	< 0.001
FBG (mmol/L)	5.1 (1.3)	5.1 (1.1)	5.0 (1.1)	5.1 (1.0)	5.1 (1.1)	< 0.001
TyG-WHtR	7.7 (1.1)	7.5 (1.1)	7.5 (1.0)	7.5 (1.0)	7.6 (1.1)	< 0.001

*MVPA*, moderate-to-vigorous physical activity; *BMI*, body mass index; *WHtR*, waist-to-height ratio; *CVD*, cardiovascular disease; *TG*, triglycerides; *FBG*, fasting blood glucose; *TyG-WHtR*, triglyceride glucose-waist height ratio.

The differences among groups were analyzed using the Chi-squared test for categorical variables, expressed as absolute frequency (%), and using one-way analysis of variance or the Kruskal–Wallis test for continuous variables, expressed as mean (standard deviation).

### Independent associations of MVPA or TyG-WHtR index with incident CVD and all-cause mortality

During a median follow-up time of 13.6 years (mean, 12.9 years; 3,875,105 person-years), 27,342 CVD cases were recorded. During a median follow-up of 13.8 years (mean, 13.5 years; 4,056,806 person-years), 21,258 deaths were recorded. Independent associations of MVPA with incident CVD and all-cause mortality are shown in **Table 2**. Compared with the < 150-min/week MVPA group, the other three groups (≥ 150 min/week MVPA) were associated with lower risks of CVD incidence and all-cause mortality. When MVPA was above 150 min/week, the HRs (95% CIs) for CVD incidence were 0.89 (0.86–0.93) in the 150–299-min/week MVPA group, 0.88 (0.85–0.91) in the 300–599-min/week MVPA group, and 0.92 (0.89–0.95) in the ≥ 600-min/week MVPA group (*p* for trend < 0.001). Similarly, the HRs (95% CIs) for all-cause mortality were 0.85 (0.81–0.88), 0.81 (0.78–0.84), and 0.82 (0.79–0.85), respectively (*p* for trend < 0.001). Additionally, the dose–response relationship between MVPA levels and incident CVD was reverse J-shaped, with a cutoff point at 261.71 min/week, whereas the association with all-cause mortality was L-shaped, leveling off up to 217.00 min/week (all overall *p* < 0.001, all nonlinear *p* < 0.001) (**Supplementary Figure S2**).

**Table 2:** Independent associations of MVPA with incident CVD and all-cause mortality.

MVPA groups	CVD				All-cause mortality			
	Incidence/person-years	Incidence rate/1,000 person-year (95% CI)	Unadjusted HR (95% CI)	Adjusted HR (95% CI)	Incidence/person-years	Incidence rate/1,000 person-year (95% CI)	Unadjusted HR (95% CI)	Adjusted HR (95% CI)
< 150 min/week	11,207/1,496,109	7.49 (7.35–7.63)	1.00 (Reference)	1.00 (Reference)	9,142/1,569,626	5.82 (5.71–5.94)	1.00 (Reference)	1.00 (Reference)
150–299 min/week	4,333/673,757	6.43 (6.24–6.62)	0.86 (0.83–0.89)	0.89 (0.86–0.93)	3,346/703,111	4.76 (4.60–4.92)	0.82 (0.78–0.85)	0.85 (0.81–0.88)
300–599 min/week	5,107/798,950	6.39 (6.22–6.57)	0.85 (0.82–0.88)	0.88 (0.85–0.91)	3,825/834,193	4.59 (4.44–4.73)	0.79 (0.76–0.82)	0.81 (0.78–0.84)
≥ 600 min/week	6,695/906,289	7.39 (7.21–7.57)	0.99 (0.96–1.02)	0.92 (0.89–0.95)	4,945/949,875	5.21 (5.06–5.35)	0.90 (0.87–0.93)	0.82 (0.79–0.85)
			*p* trend = 0.860	*p* trend < 0.001			*p* trend < 0.001	*p* trend < 0.001

*MVPA*, moderate-to-vigorous physical activity; *CVD*, cardiovascular disease; *HR*, hazard ratio; *CI*, confidence interval.

The multivariable-adjusted model was adjusted for age, sex, race, education level, Townsend deprivation index, smoking status, drinking status, parental history of CVD, self-reported use of antihypertensive drugs, self-reported use of lipid-lowering drugs, and self-reported use of insulin.

Independent associations of the TyG-WHtR index with incident CVD and all-cause mortality are shown in **Table 3**. Each additional unit of the TyG-WHtR index was associated with a 25% higher risk of CVD (HR, 1.25; 95% CI, 1.23–1.27) and a 15% higher risk of all-cause mortality (HR, 1.15; 95% CI, 1.14–1.17). Compared with TyG-WHtR tertile 1, higher TyG-WHtR tertiles were associated with increased risks of CVD incidence and all-cause mortality. The HRs (95% CIs) for CVD in TyG-WHtR tertile 2 and tertile 3 were 1.34 (1.29–1.39) and 1.72 (1.65–1.78), respectively (*p* for trend < 0.001). Similarly, the HRs (95% CIs) for all-cause mortality in TyG-WHtR tertile 2 and tertile 3 were 1.07 (1.03–1.11) and 1.29 (1.23–1.34), respectively (*p* for trend < 0.001). Additionally, the RCS regression model revealed positive nonlinear dose–response relationships of the TyG-WHtR index with CVD and all-cause mortality (all overall *p* < 0.001, all nonlinear *p* < 0.001) (**Supplementary Figure S2**).

**Table 3:** Independent associations of TyG-WHtR index with incident CVD and all-cause mortality.

TyG-WHtR	CVD				All-cause mortality			
	Incidence/person-years	Incidence rate/1,000 person-year (95% CI)	Unadjusted HR (95% CI)	Adjusted HR (95% CI)	Incidence/person-years	Incidence rate/1,000 person-year (95% CI)	Unadjusted HR (95% CI)	Adjusted HR (95% CI)
TyG-WHtR (per unit)	27,342/3,875,105	7.06 (6.97–7.14)	1.48 (1.46–1.49)	1.25 (1.23–1.27)	21,258/4,056,806	5.24 (5.17–5.31)	1.38 (1.37–1.40)	1.15 (1.14–1.17)
TyG-WHtR (tertiles)								
Q1	4,717/1,328,122	3.55 (3.45–3.65)	1.00 (Reference)	1.00 (Reference)	4,490/1,367,959	3.28 (3.19–3.38)	1.00 (Reference)	1.00 (Reference)
Q2	8,762/1,295,429	6.76 (6.62–6.91)	1.92 (1.85–1.99)	1.34 (1.29–1.39)	6,754/1,353,739	4.99 (4.87–5.11)	1.53 (1.47–1.59)	1.07 (1.03–1.11)
Q3	13,863/1,251,554	11.08 (10.89–11.26)	3.17 (3.07–3.27)	1.72 (1.65–1.78)	10,014/1,335,108	7.50 (7.35–7.65)	2.31 (2.23–2.39)	1.29 (1.23–1.34)
			*p* trend < 0.001	*p* trend < 0.001			*p* trend < 0.001	*p* trend < 0.001

*TyG-WHtR*, triglyceride glucose-waist height ratio; *CVD*, cardiovascular disease; *HR*, hazard ratio; *CI*, confidence interval.

The multivariable-adjusted model was adjusted for age, sex, race, education level, Townsend deprivation index, smoking status, drinking status, MVPA, parental history of CVD, self-reported use of antihypertensive drugs, self-reported use of lipid-lowering drugs, and self-reported use of insulin.

### Interaction analysis of MVPA and TyG-WHtR index with incident CVD and all-cause mortality

**Figure 1:**
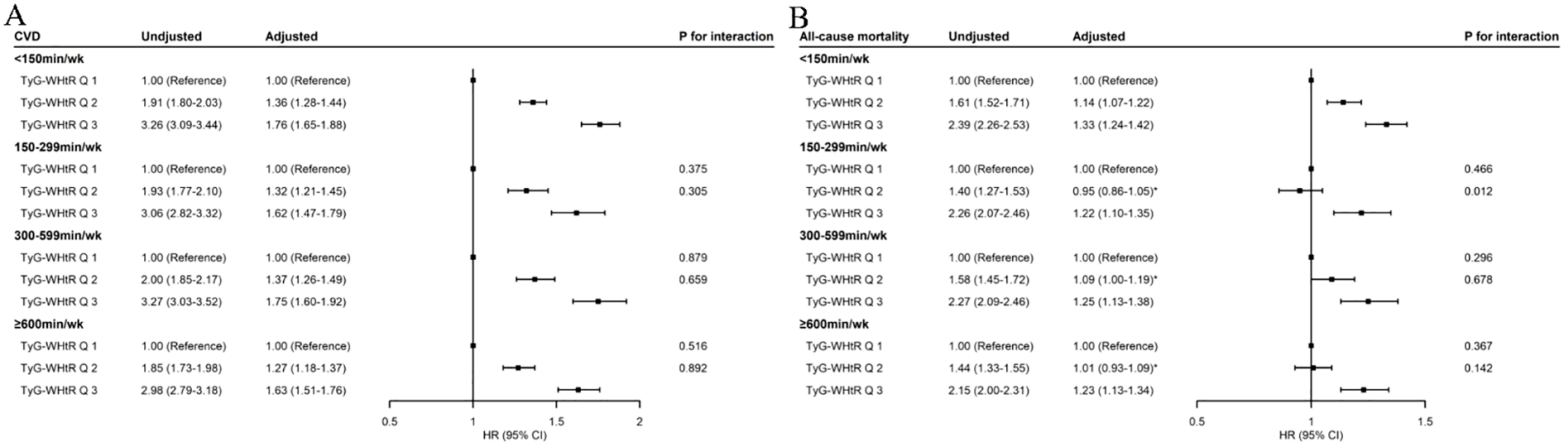
Associations of TyG-WHtR index with incident CVD **(A)** and all-cause mortality **(B)** by MVPA. MVPA, moderate-to-vigorous physical activity; TyG-WHtR, triglyceride–glucose–waist-to-height ratio; CVD, cardiovascular disease; HR, hazard ratio; CI, confidence interval. The *p*-value for interaction was obtained by including the product term of TyG-WHtR index (tertiles) and MVPA (four groups) in the multivariable-adjusted model, in which TyG-WHtR Q3 and < 150 min/week MVPA was set as the reference group. The multivariable-adjusted model was adjusted for age, sex, race, education level, Townsend deprivation index, smoking status, drinking status, parental history of CVD, self-reported use of antihypertensive drugs, self-reported use of lipid-lowering drugs, and self-reported use of insulin. ^*^Value indicates no statistical significance.

Compared to TyG-WHtR tertile 1, higher TyG-WHtR tertiles were associated with increased risks of CVD, which were not substantially different among participants of various MVPA groups (**Figure 1**). The HRs (95% CIs) for those with TyG-WHtR tertile 2 were 1.36 (1.28–1.44) in the < 150-min/week MVPA group, 1.32 (1.21–1.45) in the 150–299-min/week MVPA group, 1.37 (1.26–1.49) in the 300–599-min/week MVPA group, and 1.27 (1.18–1.37) in the ≥ 600-min/week MVPA group. Similarly, the HRs (95% CIs) for those with TyG-WHtR tertile 3 were 1.76 (1.65–1.88), 1.62 (1.47–1.79), 1.75 (1.60–1.92), and 1.63 (1.51–1.76), respectively. Additionally, the dose–response relationships between the TyG-WHtR index and CVD were consistently positive across various MVPA groups (**Supplementary Figure S3**). Compared with the < 150-min/week MVPA and TyG-WHtR tertile 3, no significant interaction was found between MVPA and the TyG-WHtR index on incident CVD (all *p* for interaction > 0.05) (**Figure 1**; **Supplementary Table S3**).

When MVPA was < 150 min/week, elevated TyG-WHtR levels were positively associated with an increased risk of all-cause mortality (**Supplementary Figure S3**). Compared with TyG-WHtR tertile 1, the HRs (95% CIs) were 1.14 (1.07–1.22) for TyG-WHtR tertile 2 and 1.33 (1.24–1.42) for TyG-WHtR tertile 3 (**Figure 1**). When MVPA achieved above 150 min/week, no significant association was found between TyG-WHtR tertile 2 and all-cause mortality, with HRs (95% CIs) of 0.95 (0.86–1.05) in the 150–299-min/week MVPA group, 1.09 (1.00–1.19) in the 300–599-min/week MVPA group, and 1.01 (0.93–1.09) in the ≥ 600-min/week MVPA group (**Figure 1**). The dose–response relationships between the TyG-WHtR index and all-cause mortality were nearly reverse L-shaped in the ≥ 150-min/week MVPA groups, with cutoff points at about 8.00, beyond which the risk increased significantly (TyG-WHtR tertile 2 range, 7.01–8.03) (**Supplementary Figure S3**). In addition, an interaction effect was found between TyG-WHtR tertile 2 and the 150–299-min/week MVPA on all-cause mortality (HR for interaction, 0.89; 95% CI, 0.81–0.97; *p* for interaction = 0.012) (**Figure 1**; **Supplementary Table S4**). However, TyG-WHtR tertile 3 was also associated with an increased risk of all-cause mortality, regardless of MVPA levels (**Figure 1**). The HRs (95% CIs) for those with TyG-WHtR tertile 3 were 1.33 (1.24–1.42) in the < 150-min/week MVPA group, 1.22 (1.10–1.35) in the 150–299-min/week MVPA group, 1.25 (1.13–1.38) in the 300–599-min/week MVPA group, and 1.23 (1.13–1.34) in the ≥ 600-min/week MVPA group.

### Joint associations of MVPA and TyG-WHtR index with incident CVD and all-cause mortality

**Figure 2:**
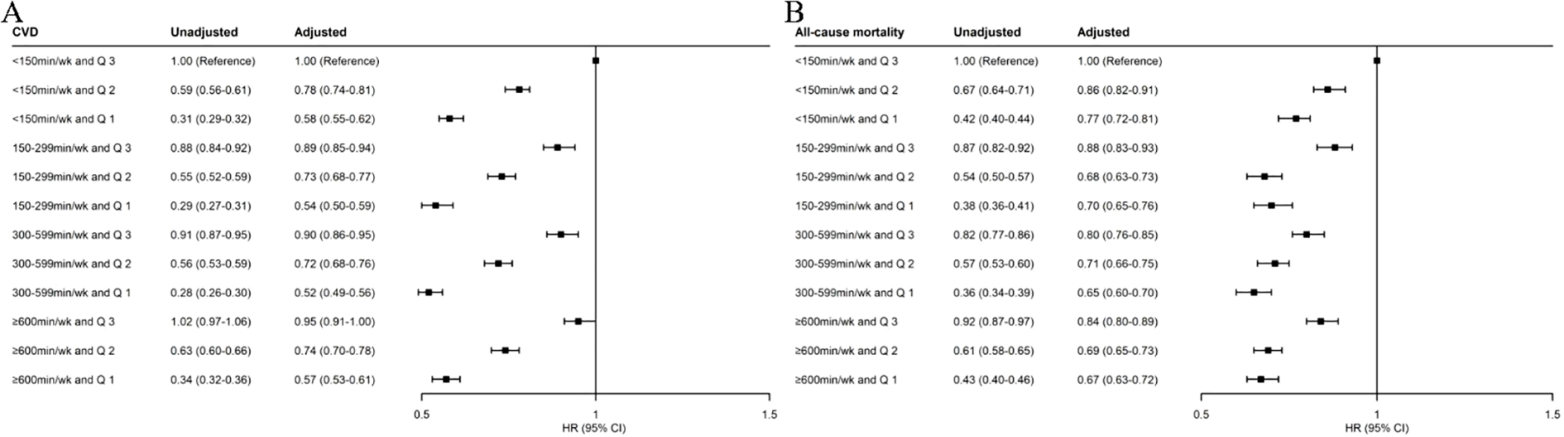
Joint associations of MVPA and TyG-WHtR index with incident CVD **(A)** and all-cause mortality **(B)**. MVPA, moderate-to-vigorous physical activity; TyG-WHtR, triglyceride–glucose–waist-to-height ratio; CVD, cardiovascular disease; HR, hazard ratio; CI, confidence interval. The multivariable-adjusted model was adjusted for age, sex, race, education level, Townsend deprivation index, smoking status, drinking status, parental history of CVD, self-reported use of antihypertensive drugs, self-reported use of lipid-lowering drugs, and self-reported use of insulin.

Compared with the reference group (< 150 min/week MVPA and TyG-WHtR tertile 3), all other combined groups were associated with lower risks of CVD (**Figure 2**). The HRs (95% CIs) of CVD incidence for TyG-WHtR tertile 3 were 1.00 (reference) in the < 150-min/week MVPA group, 0.89 (0.85–0.94) in the 150–299-min/week MVPA group, 0.90 (0.86–0.95) in the 300–599-min/week MVPA group, and 0.95 (0.91–1.00) in the ≥ 600-min/week MVPA group. The HRs (95% CIs) of CVD incidence for TyG-WHtR tertile 2 were 0.78 (0.74–0.81), 0.73 (0.68–0.77), 0.72 (0.68–0.76), and 0.74 (0.70–0.78), respectively. The HRs (95% CIs) of CVD incidence for TyG-WHtR tertile 1 were 0.58 (0.55–0.62), 0.54 (0.50–0.59), 0.52 (0.49–0.56), and 0.57 (0.53–0.61), respectively.

Compared with the reference group, all other combined groups were also associated with lower risks of all-cause mortality (**Figure 2**). The HRs (95% CIs) of all-cause mortality for TyG-WHtR tertile 3 were 1.00 (reference) in the < 150-min/week MVPA group, 0.88 (0.83–0.93) in the 150–299-min/week MVPA group, 0.80 (0.76–0.85) in the 300–599-min/week MVPA group, and 0.84 (0.80–0.89) in the ≥ 600-min/week MVPA group. The HRs (95% CIs) of all-cause mortality for TyG-WHtR tertile 2 were 0.86 (0.82–0.91), 0.68 (0.63–0.73), 0.71 (0.66–0.75), and 0.69 (0.65–0.73), respectively. The HRs (95% CIs) of all-cause mortality for TyG-WHtR tertile 1 were 0.77 (0.72–0.81), 0.70 (0.65–0.76), 0.65 (0.60–0.70), and 0.67 (0.63–0.72), respectively.

### Subgroup and sensitivity analyses

Associations of TyG-WHtR with incident CVD and all-cause mortality in each MVPA group were generally similar across sex (men and women) and education level (high and low) subgroups (**Supplementary Tables S7**; **Supplementary Tables S11**). However, the associations were different across age (< 65 and ≥ 65 years) and race (White and non-White) subgroups (**Supplementary Tables S5**; **Supplementary Tables S9**). Specifically, among participants aged < 65 years, elevated TyG-WHtR tertiles were associated with increased risks of all-cause mortality across all MVPA levels, and the association between TyG-WHtR tertile 2 and all-cause mortality remained statistically significant, with HRs (95% CIs) of 1.14 (1.00–1.29) in the 150–299-min/week group, 1.31 (1.17–1.47) in the 300–599-min/week group, and 1.20 (1.08–1.33) in the ≥ 600-min/week group (**Supplementary Table S5**). Among non-White participants, TyG-WHtR tertile 2 was not associated with CVD incidence compared with tertile 1, with HRs (95% CIs) of 1.04 (0.65–1.67) in the 150–299-min/week group and 0.92 (0.61–1.40) in the ≥ 600-min/week group (**Supplementary Table S9**).

The joint associations of MVPA and TyG-WHtR with CVD incidence and all-cause mortality appeared to show no significant differences across race and education level subgroups (**Supplementary Tables S10**; **Supplementary Tables S12**). However, the joint associations with CVD incidence were stronger in younger and female participants, with greater CVD risk reduction in younger and female participants (most *p* for interaction < 0.05) (**Supplementary Tables S6**; **Supplementary Tables S8**). For all-cause mortality, the joint associations were stronger in female participants (most *p* for interaction < 0.05), with greater mortality risk reduction in this subgroup (**Supplementary Table S8**).

The results remained similar after we excluded participants who had CVD or mortality within the first 2 years of follow-up, used multiple imputation to impute all missing covariates, further adjusted for sedentary behavior (< 6 and ≥ 6 h/day) in the multivariable-adjusted model, or repeated all analyses by substituting the TyG-WHtR index with the TyG-WC index (**Supplementary Tables S13–S27**).

## Discussion

This study demonstrated independent and joint associations of MVPA and TyG-WHtR (an IR surrogate) with incident CVD and all-cause mortality. MVPA demonstrated a reverse J-shaped association with incident CVD, with a cutoff point at 261.71 min/week, and an L-shaped association with all-cause mortality, leveling off at 217.00 min/week. Elevated TyG-WHtR was positively associated with increased risks of CVD incidence and all-cause mortality. MVPA levels did not modify the association of TyG-WHtR with CVD incidence, whereas guideline-recommended MVPA (150–299 min/week) and TyG-WHtR tertile 2 could synergistically reduce the risk of all-cause mortality. Among participants with MVPA levels beyond guideline recommendations (300–599 or ≥ 600 min/week), the extent of risk reduction for IR-related incident CVD and all-cause mortality was similar to that observed in those with guideline-recommended MVPA.

This study identified a reverse J-shaped association between MVPA levels and incident CVD, with a cutoff point at 261.71 min/week, whereas an L-shaped association was observed for all-cause mortality, with the risk plateauing at 217.00 min/week. These findings are consistent with previous studies demonstrating dose–response relationships between PA and morbidity/mortality, where risk reduction plateaus beyond certain PA thresholds (36–39). The results support existing PA guidelines indicating that 150–300 min/week of MVPA can reduce risks for multiple health outcomes, with benefits plateauing beyond this range (28, 29). In addition, our findings demonstrated that elevated TyG-WHtR was positively associated with the risks of incident CVD and all-cause mortality, which aligns with previous studies (20, 21, 24, 40–42). These findings emphasize the importance of alleviating IR, as reflected by TyG-WHtR, to improve cardiometabolic health and survival. However, current research has primarily focused on the separate relationships of the IR surrogate or PA with outcomes (20, 21, 24, 36–42). It remains unclear whether MVPA can modify the associations of IR with incident CVD and all-cause mortality, and whether MVPA levels beyond guideline recommendations could further mitigate these IR-related risks.

This study further revealed that even among individuals meeting or exceeding current MVPA recommendations, elevated TyG-WHtR remained associated with increased CVD risk. In addition, MVPA levels did not modify the associations between TyG-WHtR and incident CVD, and the positive association was not substantially different across MVPA groups. Notably, among participants with guideline-recommended MVPA (150–299 min/week), moderate IR (TyG-WHtR tertile 2) showed no significant association with all-cause mortality compared with mild IR (TyG-WHtR tertile 1). A similar lack of significant association was observed among those engaged in 300–599 or ≥ 600 min/week MVPA. These findings suggest that adherence to guideline-recommended or higher levels of MVPA may offset the mortality risk associated with moderate IR. Interestingly, a synergistic effect was observed between 150–299 min/week MVPA and moderate IR on all-cause mortality, suggesting the importance of guideline-recommended MVPA (150–299 min/week) to reduce all-cause mortality risk, particularly in individuals with moderate IR (TyG-WHtR: 7.01–8.03), who appear more susceptible to its protective effects. We also found that among participants with severe IR, meeting or exceeding MVPA recommendations (150–299, 300–599, or ≥ 600 min/week) reduced all-cause mortality risk, but it did not completely offset this risk. This suggests that promoting MVPA alone may be insufficient to fully counteract mortality risks associated with relatively severe IR, potentially requiring additional interventions such as dietary modification or pharmacotherapy (43).

Furthermore, this study found that promoting MVPA and alleviating IR were jointly associated with lower risks of incident CVD and all-cause mortality. Notably, when MVPA met or exceeded guideline recommendations, the joint associations of MVPA and IR with the risks of incident CVD and all-cause mortality were not further attenuated. Therefore, a similar reduction in the IR-related risks of incident CVD and all-cause mortality was observed among participants who met the guideline-recommended MVPA and those who exceeded it.

In subgroup analyses, stronger joint associations were observed in younger adults and female participants for incident CVD, and in female participants for all-cause mortality. Among younger participants, elevated TyG-WHtR tertiles were associated with increased risks of all-cause mortality in each MVPA subgroup, and the risks associated with TyG-WHtR tertile 2 remained statistically significant even when participants achieved or exceeded guideline-recommended MVPA. Additionally, improving IR and promoting MVPA provided greater benefits for reducing CVD risk in younger adults. A possible explanation may be the higher prevalence of multiple comorbidities and greater exposure to cardiovascular risk factors in older populations, potentially attenuating the combined effects of IR and MVPA on cardiovascular outcomes. In addition, the joint associations of MVPA and IR with incident CVD and all-cause mortality were stronger in women. The heightened metabolic vulnerability among women may be explained by estrogen’s role in modulating adiponectin and insulin sensitivity (44), and MVPA may improve IR by promoting estrogen secretion (45). However, these hypotheses require validation in future studies.

The complex effects of IR and PA on incident CVD and all-cause mortality may be explained by the following mechanisms. Elevated TyG-WHtR reflects IR, which is closely related to potential metabolic disturbances such as disordered glucolipid metabolism and visceral fat accumulation (46, 47). These disturbances can collectively promote endothelial dysfunction, chronic inflammation, and systemic oxidative stress, thereby increasing the risks of incident CVD and all-cause mortality (10, 48–51). Conversely, PA exerts protective effects on systemic metabolic regulation by improving IR (43), visceral adiposity (52), systemic inflammation (53), and mitochondrial dysfunction (54), thereby mitigating the adverse effects of IR on incident CVD and all-cause mortality. The observed reverse J- and L-shaped dose–response relationships suggest a plateauing effect, where the initial, substantial cardiometabolic benefits of increasing PA reach an optimum, beyond which additional PA provides minimal further benefit (36–39). In line with this, our findings demonstrate that the protection offered by MVPA beyond guideline recommendations was consistent with, but did not exceed, that offered by guideline-recommended MVPA in attenuating IR-related risks. In some cases of very high-volume exercise, potential mechanisms, such as mitochondrial functional impairment and decreased glucose tolerance, might counterbalance the benefits, preventing further IR-related risk reduction. Further studies are needed to explore the pathways underlying the interaction between IR and PA on health outcomes.

### Strengths and limitations

The major strengths of this study include the large sample size and long-term follow-up from a prospective cohort in the UK, which allowed us to perform the interaction and joint analyses with sufficient statistical power. We also conducted sensitivity analyses to show the robustness of the findings. Importantly, our work highlights the effects of MVPA on IR-related outcomes, which helps to conduct metabolic risk stratification and targeted interventions. The study also has certain limitations. First, MVPA trajectories and TyG-WHtR index changes could not be captured, so causal inference cannot be made, and the observed associations might be attenuated due to nondifferential misclassification bias. Future studies with repeated measurements are preferred. Second, MVPA was self-reported, and there is potential for recall or social desirability bias, which may obscure the true magnitude and nature of the association (55). Third, the participants were mostly White, which limits the generalizability of our findings to other populations. The number of participants and events might be insufficient in the non-White subgroup, and the results should be interpreted with caution. Finally, given the nature of observational studies, while we adjusted for some potential confounding factors as much as possible and conducted subgroup analyses, the potential influence of unmeasured confounders such as diet quality, medication adherence, and psychological stress remains possible and should be acknowledged as residual confounding.

## Conclusions

Promoting MVPA and improving IR could independently and jointly mitigate the risks of incident CVD and all-cause mortality. A synergistic effect between guideline-recommended MVPA (150–299 min/week) and moderate IR (TyG-WHtR: 7.01–8.03) on all-cause mortality highlights the importance of promoting MVPA to reduce all-cause mortality risk, particularly among individuals with moderate IR, who are more susceptible to its protective effects. Furthermore, the effects of exceeding the guideline-recommended MVPA (300–599 and ≥ 600 min/week) in attenuating the deleterious associations of IR with incident CVD and all-cause mortality were consistent with those of meeting the guideline-recommended level.

## Data Availability

Publicly available datasets were analyzed in this study. This data can be found here: The UK Biobank data are available directly from UK Biobank upon submission of a data request proposal (www.ukbiobank.ac.uk/).

## Supplementary material

Supplementary Table 1 Baseline characteristics of participants according to the tertiles of the TyG-WHtR index.

Supplementary Table 2 Baseline characteristics of participants excluded and included in the analysis.

Supplementary Table 3 Interaction between MVPA and TyG-WHtR on incident CVD.

Supplementary Table 4 Interaction between MVPA and TyG-WHtR on all-cause mortality.

Supplementary Table 5 Associations of TyG-WHtR index with incident CVD and all-cause mortality by MVPA in age subgroups.

Supplementary Table 6 Joint associations of MVPA and TyG-WHtR with incident CVD and all-cause mortality in age subgroups.

Supplementary Table 7 Associations of TyG-WHtR index with incident CVD and all-cause mortality by MVPA in sex subgroups.

Supplementary Table 8 Joint associations of MVPA and TyG-WHtR with incident CVD and all-cause mortality in sex subgroups.

Supplementary Table 9 Associations of TyG-WHtR index with incident CVD and all-cause mortality by MVPA in race subgroups.

Supplementary Table 10 Joint associations of MVPA and TyG-WHtR with incident CVD and all-cause mortality in race subgroups.

Supplementary Table 11 Associations of TyG-WHtR index with incident CVD and all-cause mortality by MVPA in education level subgroups.

Supplementary Table 12 Joint associations of MVPA and TyG-WHtR with incident CVD and all-cause mortality in education level subgroups.

Supplementary Table 13 Independent associations of MVPA with incident CVD and all-cause mortality after excluding participants who had outcomes within the first two years of follow-up.

Supplementary Table 14 Independent associations of TyG-WHtR index with incident CVD and all-cause mortality after excluding participants who had outcomes within the first two years of follow-up.

Supplementary Table 15 Associations of TyG-WHtR index with incident CVD and all-cause mortality by MVPA after excluding participants who had outcomes within the first two years of follow-up.

Supplementary Table 16 Joint associations of MVPA and TyG-WHtR with incident CVD and all-cause mortality after excluding participants who had outcomes within the first two years of follow-up.

Supplementary Table 17 Independent associations of MVPA with incident CVD and all-cause mortality after imputing all missing covariates.

Supplementary Table 18 Independent associations of TyG-WHtR index with incident CVD and all-cause mortality after imputing all missing covariates.

Supplementary Table 19 Associations of TyG-WHtR index with incident CVD and all-cause mortality by MVPA after imputing all missing covariates.

Supplementary Table 20 Joint associations of MVPA and TyG-WHtR with incident CVD and all-cause mortality after imputing all missing covariates.

Supplementary Table 21 Independent associations of MVPA with incident CVD and all-cause mortality after further adjusting sedentary behavior in multivariable-adjusted model.

Supplementary Table 22 Independent associations of TyG-WHtR index with incident CVD and all-cause mortality after further adjusting sedentary behavior in multivariable-adjusted model.

Supplementary Table 23 Associations of TyG-WHtR index with incident CVD and all-cause mortality by MVPA after further adjusting sedentary behavior in multivariable-adjusted model.

Supplementary Table 24 Joint associations of MVPA and TyG-WHtR with incident CVD and all-cause mortality after further adjusting sedentary behavior in multivariable-adjusted model.

Supplementary Table 25 Independent associations of TyG-WC index with incident CVD and all-cause mortality.

Supplementary Table 26 Associations of TyG-WC index with incident CVD and all-cause mortality by MVPA.

Supplementary Table 27 Joint associations of MVPA and TyG-WC with incident CVD and all-cause mortality Baseline characteristics of participants according to the tertiles of TyG-WHtR index.

Supplementary Figure 1 Flowchart of participant screening.

Supplementary Figure 2 Dose-response associations of MVPA (A, B) and TyG-WHtR index (C, D) with incident CVD and all-cause mortality.

Supplementary Figure 3 Dose-response associations of TyG-WHtR index with incident CVD (A-D) and all-cause mortality (E-H) stratified by MVPA
