# Supplementary material for: Effects of moderate-to-vigorous physical activity on the associations between an insulin resistance surrogate and incident cardiovascular disease and all-cause mortality: a UK Biobank cohort study
Source: Front Endocrinol (Lausanne). 2025 Nov 5;16:1709135. doi: 10.3389/fendo.2025.1709135 (PMC12626858; doi:10.3389/fendo.2025.1709135)
Supplement: Supplementary Table 1 — Baseline characteristics of participants according to the tertiles of the TyG-WHtR index. [file DataSheet1.docx]

**Supplementary material**

Table S1. Baseline characteristics of participants according to the tertiles of TyG-WHtR index

Table S2. Baseline characteristics of participants excluded and included in the analysis

Table S3. Interaction between MVPA and TyG-WHtR on incident CVD

Table S4. Interaction between MVPA and TyG-WHtR on all-cause mortality

Table S5. Associations of TyG-WHtR index with incident CVD and all-cause mortality by MVPA in age subgroups

Table S6. Joint associations of MVPA and TyG-WHtR with incident CVD and all-cause mortality in age subgroups

Table S7. Associations of TyG-WHtR index with incident CVD and all-cause mortality by MVPA in sex subgroups

Table S8. Joint associations of MVPA and TyG-WHtR with incident CVD and all-cause mortality in sex subgroups

Table S9. Associations of TyG-WHtR index with incident CVD and all-cause mortality by MVPA in race subgroups

Table S10. Joint associations of MVPA and TyG-WHtR with incident CVD and all-cause mortality in race subgroups

Table S11. Associations of TyG-WHtR index with incident CVD and all-cause mortality by MVPA in education level subgroups

Table S12. Joint associations of MVPA and TyG-WHtR with incident CVD and all-cause mortality in education level subgroups

Table S13. Independent associations of MVPA with incident CVD and all-cause mortality after excluding participants who had outcomes within the first two years of follow-up

Table S14. Independent associations of TyG-WHtR index with incident CVD and all-cause mortality after excluding participants who had outcomes within the first two years of follow-up

Table S15. Associations of TyG-WHtR index with incident CVD and all-cause mortality by MVPA after excluding participants who had outcomes within the first two years of follow-up

Table S16. Joint associations of MVPA and TyG-WHtR with incident CVD and all-cause mortality after excluding participants who had outcomes within the first two years of follow-up

Table S17. Independent associations of MVPA with incident CVD and all-cause mortality after imputing all missing covariates

Table S18. Independent associations of TyG-WHtR index with incident CVD and all-cause mortality after imputing all missing covariates

Table S19. Associations of TyG-WHtR index with incident CVD and all-cause mortality by MVPA after imputing all missing covariates

Table S20. Joint associations of MVPA and TyG-WHtR with incident CVD and all-cause mortality after imputing all missing covariates

Table S21. Independent associations of MVPA with incident CVD and all-cause mortality after further adjusting sedentary behavior in multivariable-adjusted model

Table S22. Independent associations of TyG-WHtR index with incident CVD and all-cause mortality after further adjusting sedentary behavior in multivariable-adjusted model

Table S23. Associations of TyG-WHtR index with incident CVD and all-cause mortality by MVPA after further adjusting sedentary behavior in multivariable-adjusted model

Table S24. Joint associations of MVPA and TyG-WHtR with incident CVD and all-cause mortality after further adjusting sedentary behavior in multivariable-adjusted model

Table S25. Independent associations of TyG-WC index with incident CVD and all-cause mortality

Table S26. Associations of TyG-WC index with incident CVD and all-cause mortality by MVPA

Table S27. Joint associations of MVPA and TyG-WC with incident CVD and all-cause mortality

Fig. S1. Flowchart of participant screening

Fig. S2. Dose-response associations of MVPA (A, B) and TyG-WHtR index (C, D) with incident CVD and all-cause mortality

Fig. S3. Dose-response associations of TyG-WHtR index with incident CVD (A-D) and all-cause mortality (E-H) stratified by MVPA

| **Table S1. Baseline characteristics of participants according to the tertiles of TyG-WHtR index** | | | | | |
| --- | --- | --- | --- | --- | --- |
| **Variables** | **TyG-BMI tertile 1** | **TyG-BMI tertile 2** | **TyG-BMI tertile 3** | **Total** | ***P* value** |
|  | **(≤ 7.01)** | **(7.01-8.03)** | **(> 8.03)** |  |  |
| Number | 99976 | 99976 | 99976 | 299928 |  |
| Age (yrs) | 54.2(8.1) | 56.4(8.0) | 57.1(7.8) | 55.9(8.0) | <0.001 |
| Sex (%) |  |  |  |  | <0.001 |
| Male | 11392(11.4) | 46284(46.3) | 79517(79.5) | 137193(45.7) |  |
| Female | 88584(88.6) | 53692(53.7) | 20459(20.5) | 162735(54.3) |  |
| Race (%) |  |  |  |  | <0.001 |
| White | 95757(95.8) | 95515(95.5) | 95642(95.7) | 286914(95.7) |  |
| Black | 1557(1.6) | 1431(1.4) | 720(0.7) | 3708(1.2) |  |
| Asian | 1275(1.3) | 1808(1.8) | 2440(2.4) | 5523(1.8) |  |
| Other | 1387(1.4) | 1222(1.2) | 1174(1.2) | 3783(1.3) |  |
| Educational level (%) |  |  |  |  | <0.001 |
| High | 43841(43.9) | 48352(48.4) | 52115(52.1) | 144308(48.1) |  |
| low | 56135(56.1) | 51624(51.6) | 47861(47.9) | 155620(51.9) |  |
| Townsend deprivation index (%) | |  |  |  | <0.001 |
| Most deprived | 15989(16.0) | 17276(17.3) | 18542(18.5) | 51807(17.3) |  |
| Intermediate deprived | 61515(61.5) | 60912(60.9) | 60846(60.9) | 183273(61.1) |  |
| Least deprived | 22472(22.5) | 21788(21.8) | 20588(20.6) | 64848(21.6) |  |
| BMI (kg/m^2^) | 24.7(3.9) | 27.2(4.4) | 29.5(4.3) | 27.2(4.6) | <0.001 |
| WHtR | 0.8(0.0) | 0.9(0.0) | 1.0(0.1) | 0.9(0.1) | <0.001 |
| Current smoking (%) |  |  |  |  | <0.001 |
| No | 92677(92.7) | 90539(90.6) | 87989(88.0) | 271205(90.4) |  |
| Yes | 7299(7.3) | 9437(9.4) | 11987(12.0) | 28723(9.6) |  |
| Moderate drinking (%) |  |  |  |  | <0.001 |
| No | 60970(61.0) | 57312(57.3) | 56731(56.7) | 175013(58.4) |  |
| Yes | 39006(39.0) | 42664(42.7) | 43245(43.3) | 124915(41.6) |  |
| MVPA (min/wk) | 439.7(552.9) | 441.2(585.3) | 408.8(601.2) | 429.89(580.4) | <0.001 |
| Parental history of CVD (%) | |  |  |  | <0.001 |
| No | 44648(44.7) | 42302(42.3) | 42284(42.3) | 129234(43.1) |  |
| Yes | 55328(55.3) | 57674(57.7) | 57692(57.7) | 170694(56.9) |  |
| Self-reported use of antihypertensive drugs (%) | |  |  |  | <0.001 |
| No | 91521(91.5) | 83547(83.6) | 73896(73.9) | 248964(83.0) |  |
| Yes | 8455(8.5) | 16429(16.4) | 26080(26.1) | 50964(17.0) |  |
| Self-reported use of lipid-lowering drugs (%) | |  |  |  | <0.001 |
| No | 94768(94.8) | 88026(88.0) | 78544(78.6) | 261338(87.1) |  |
| Yes | 5208(5.2) | 11950(12.0) | 21432(21.4) | 38590(12.9) |  |
| Self-reported use of insulin (%) |  |  |  |  | <0.001 |
| No | 99557(99.6) | 99408(99.4) | 98476(98.5) | 297441(99.2) |  |
| Yes | 419(0.4) | 568(0.6) | 1500(1.5) | 2487(0.8) |  |
| TG (mmol/L) | 1.1(0.4) | 1.6(0.6) | 2.5(1.2) | 1.7(1.0) | <0.001 |
| FBG (mmol/L) | 4.8(0.6) | 5.0(0.8) | 5.4(1.6) | 5.1(1.1) | <0.001 |
| TyG-WHtR | 6.4(0.4) | 7.5(0.3) | 8.8(0.6) | 7.6(1.1) | <0.001 |

MVPA, moderate to vigorous physical activity; BMI, body mass index; WHtR, waist height ratio; CVD, cardiovascular disease; TG, triglycerides; FBG, fasting blood glucose; TyG-WHtR, triglyceride glucose-waist height ratio.

The differences among groups were analyzed using the Chi-squared test for categorical variables, expressed as absolute frequency (%), and using one-way analysis of variance or Kruskal–Wallis test for continuous variables, expressed as mean (standard deviation).

| **Table S2. Baseline characteristics of participants excluded and included in the analysis** | | | |
| --- | --- | --- | --- |
| **Variables** | **Exclude** | **Include** | ***P* value** |
|  | **(n=202428)** | **(n=299928)** |  |
| Age (yrs) | 57.4(8.1) | 55.9(8.0) | <0.001 |
| Sex (%) |  |  | 0.012 |
| Male | 91869(45.4) | 137193(45.7) |  |
| Female | 110559(54.6) | 162735(54.3) |  |
| Race (%) |  |  | <0.001 |
| White | 185647(93.0) | 286914(95.7) |  |
| Black | 4350(2.2) | 3708(1.2) |  |
| Asian | 5928(3.0) | 5523(1.8) |  |
| Other | 3725(1.9) | 3783(1.3) |  |
| Educational level (%) |  |  | <0.001 |
| High | 119899(60.6) | 144308(48.1) |  |
| Low | 77887(39.4) | 155620(51.9) |  |
| Townsend deprivation index (%) |  |  | <0.001 |
| Most deprived | 48533(24.0) | 51807(17.3) |  |
| Intermediate deprived | 117489(58.2) | 183273(61.1) |  |
| Least deprived | 35780(17.7) | 64848(21.6) |  |
| BMI (kg/m^2^) | 27.8(5.0) | 27.2(4.6) | <0.001 |
| WHtR | 0.9(0.1) | 0.9(0.1) | <0.001 |
| Current smoking (%) |  |  | <0.001 |
| No | 175243(87.9) | 271205(90.4) |  |
| Yes | 24235(12.1) | 28723(9.6) |  |
| Moderate drinking (%) |  |  | <0.001 |
| No | 105160(52.3) | 175013(58.4) |  |
| Yes | 95766(47.7) | 124915(41.6) |  |
| MVPA (min/wk) | 538.6(1020.9) | 429.89(580.4) | <0.001 |
| Parental history of CVD (%) | |  | <0.001 |
| No | 62529(38.9) | 129234(43.1) |  |
| Yes | 98060(61.1) | 170694(56.9) |  |
| Self-reported use of antihypertensive drugs (%) | |  | <0.001 |
| No | 140809(72.6) | 248964(83.0) |  |
| Yes | 53014(27.4) | 50964(17.0) |  |
| Self-reported use of lipid-lowering drugs (%) | |  | <0.001 |
| No | 145541(75.1) | 261338(87.1) |  |
| Yes | 48282(24.9) | 38590(12.9) |  |
| Self-reported use of insulin (%) |  |  | <0.001 |
| No | 190699(98.4) | 297441(99.2) |  |
| Yes | 3124(1.6) | 2487(0.8) |  |
| TG (mmol/L) | 1.8(1.0) | 1.7(1.0) | <0.001 |
| FBG (mmol/L) | 5.2(1.4) | 5.1(1.1) | <0.001 |
| TyG-WHtR | 7.8(1.1) | 7.6(1.1) | <0.001 |

MVPA, moderate to vigorous physical activity; BMI, body mass index; WHtR, waist height ratio; CVD, cardiovascular disease; TG, triglycerides; FBG, fasting blood glucose; TyG-WHtR, triglyceride glucose-waist height ratio.

The differences among groups were analyzed using the Chi-squared test for categorical variables, expressed as absolute frequency (%), and using one-way analysis of variance or Kruskal–Wallis test for continuous variables, expressed as mean (standard deviation).

| **Table S3. Interaction between MVPA and TyG-WHtR on incident CVD** | | | |
| --- | --- | --- | --- |
|  | **Adjusted HR (95% CI)** | | |
|  | **TyG-WHtR Q 3** | **TyG-WHtR Q 2** | **TyG-WHtR Q 1** |
| **<150 min/wk MVPA** | 1.00 (Reference) | 1.00 (Reference) | 1.00 (Reference) |
| **150-299 min/wk MVPA** | 1.00 (Reference) | 1.04 (0.96-1.13) | 1.05 (0.95-1.15) |
| **300-599 min/wk MVPA** | 1.00 (Reference) | 1.02 (0.94-1.10) | 0.99 (0.91-1.09) |
| **≥600 min/wk MVPA** | 1.00 (Reference) | 1.00 (0.93-1.07) | 1.03 (0.95-1.12) |

MVPA, moderate to vigorous physical activity; TyG-WHtR, triglyceride glucose-waist height ratio; CVD, cardiovascular disease; HR, hazard ratio; CI, confidence interval.

The product term of TyG-WHtR index (tertiles) and MVPA (four groups) was included in the multivariable-adjusted model and the HR (95%CI) of the product term was the measure of interaction on the multiplicative scale.

The multivariable-adjusted model was adjusted for age, sex, race, education level, Townsend deprivation index, smoking status, drinking status, parental history of CVD, self-reported use of antihypertensive drugs, self-reported use of lipid-lowering drugs and self-reported use of insulin.

| **Table S4. Interaction between MVPA and TyG-WHtR on all-cause mortality** | | | |
| --- | --- | --- | --- |
|  | **Adjusted HR (95% CI)** | | |
|  | **TyG-WHtR Q 3** | **TyG-WHtR Q 2** | **TyG-WHtR Q 1** |
| **<150 min/wk MVPA** | 1.00 (Reference) | 1.00 (Reference) | 1.00 (Reference) |
| **150-299 min/wk MVPA** | 1.00 (Reference) | **0.89 (0.81-0.97)** | 1.04 (0.94-1.15) |
| **300-599 min/wk MVPA** | 1.00 (Reference) | 1.02 (0.93-1.11) | 1.05 (0.95-1.16) |
| **≥600 min/wk MVPA** | 1.00 (Reference) | 0.94 (0.87-1.02) | 1.04 (0.95-1.14) |

MVPA, moderate to vigorous physical activity; TyG-WHtR, triglyceride glucose-waist height ratio; CVD, cardiovascular disease; HR, hazard ratio; CI, confidence interval.

The product term of TyG-WHtR index (tertiles) and MVPA (four groups) was included in the multivariable-adjusted model and the HR (95%CI) of the product term was the measure of interaction on the multiplicative scale.

The multivariable-adjusted model was adjusted for age, sex, race, education level, Townsend deprivation index, smoking status, drinking status, parental history of CVD, self-reported use of antihypertensive drugs, self-reported use of lipid-lowering drugs and self-reported use of insulin.

| **Table S5. Associations of TyG-WHtR index with incident CVD and all-cause mortality by MVPA in age subgroups** | | | | | | | | |
| --- | --- | --- | --- | --- | --- | --- | --- | --- |
|  | **CVD** | | | | **All-cause mortality** | | | |
|  | **Age<65(n=250817)** | | **Age≥65(n=49111)** | | **Age<65(n=250817)** | | **Age≥65(n=49111)** | |
|  | **Adjusted HR (95% CI)** | ***P* for interaction** | **Adjusted HR (95% CI)** | ***P* for interaction** | **Adjusted HR (95% CI)** | ***P* for interaction** | **Adjusted HR (95% CI)** | ***P* for interaction** |
| <150min/wk |  |  |  |  |  |  |  |  |
| TyG-WHtR Q 1 | 1.00 (Reference) |  | 1.00 (Reference) |  | 1.00 (Reference) |  | 1.00 (Reference) |  |
| TyG-WHtR Q 2 | 1.55 (1.44-1.67) |  | 1.17 (1.04-1.31) |  | 1.31 (1.22-1.42) |  | 1.11 (0.99-1.24) |  |
| TyG-WHtR Q 3 | 2.10 (1.94-2.26) |  | 1.43 (1.27-1.61) |  | 1.60 (1.47-1.74) |  | 1.23 (1.09-1.39) |  |
| 150-299min/wk |  |  |  |  |  |  |  |  |
| TyG-WHtR Q 1 | 1.00 (Reference) | 0.248 | 1.00 (Reference) | 0.711 | 1.00 (Reference) | 0.705 | 1.00 (Reference) | 0.324 |
| TyG-WHtR Q 2 | 1.45 (1.29-1.62) | 0.517 | 1.31 (1.12-1.53) | 0.385 | **1.14 (1.00-1.29)** | 0.077 | 0.90 (0.76-1.06) | 0.121 |
| TyG-WHtR Q 3 | 1.85 (1.64-2.09) |  | 1.53 (1.29-1.82) |  | 1.51 (1.32-1.73) |  | 1.23 (0.95-1.34) |  |
| 300-599min/wk |  |  |  |  |  |  |  |  |
| TyG-WHtR Q 1 | 1.00 (Reference) | 0.509 | 1.00 (Reference) | 0.924 | 1.00 (Reference) | 0.730 | 1.00 (Reference) | 0.251 |
| TyG-WHtR Q 2 | 1.59 (1.44-1.77) | 0.709 | 1.24 (1.08-1.43) | 0.315 | **1.31 (1.17-1.47)** | 0.965 | 1.05 (0.90-1.22) | 0.541 |
| TyG-WHtR Q 3 | 2.22 (1.99-2.48) |  | 1.42 (1.22-1.66) |  | 1.61 (1.42-1.82) |  | 1.14 (0.97-1.34) |  |
| ≥600min/wk |  |  |  |  |  |  |  |  |
| TyG-WHtR Q 1 | 1.00 (Reference) | 0.398 | 1.00 (Reference) | 0.482 | 1.00 (Reference) | 0.112 | 1.00 (Reference) | 0.980 |
| TyG-WHtR Q 2 | 1.52 (1.39-1.66) | 0.755 | 1.09 (0.96-1.23) | 0.380 | **1.20 (1.08-1.33)** | 0.930 | 0.97 (0.85-1.10) | 0.041 |
| TyG-WHtR Q 3 | 2.06 (1.87-2.26) |  | 1.33 (1.17-1.51) |  | 1.48 (1.32-1.65) |  | 1.25 (1.09-1.43) |  |

MVPA, moderate to vigorous physical activity; TyG-WHtR, triglyceride glucose-waist height ratio; CVD, cardiovascular disease; HR, hazard ratio; CI, confidence interval.

The P value for interaction was obtained including the product term of TyG-WHtR index (tertiles) and MVPA (four groups) in the multivariable-adjusted model, among which TyG-WHtR Q 3 and <150min/wk MVPA was set as the reference group.

The multivariable-adjusted model was adjusted for sex, race, education level, Townsend deprivation index, smoking status, drinking status, parental history of CVD, self-reported use of antihypertensive drugs, self-reported use of lipid-lowering drugs and self-reported use of insulin.

| **Table S6. Joint associations of MVPA and TyG-WHtR with incident CVD and all-cause mortality in age subgroups** | | | | | | | | | |  |
| --- | --- | --- | --- | --- | --- | --- | --- | --- | --- | --- |
|  | **CVD** | | | | | **All-cause mortality** | | | |  |
|  | **Adjusted HR (95% CI)** | | ***P* for interaction** | | | **Adjusted HR (95% CI)** | | ***P* for interaction** | |  |
|  | **Age<65(n=250817)** | **Age≥65(n=49111)** | |  | **Age<65(n=250817)** | | **Age≥65(n=49111)** | |  | |
| <150 min/wk MVPA and TyG-WHtR Q 3 | 1.00 (Reference) | 1.00 (Reference) | |  | 1.00 (Reference) | | 1.00 (Reference) | |  | |
| <150 min/wk MVPA and TyG-WHtR Q 2 | 0.74 (0.70-0.78) | 0.83 (0.77-0.90) | | **<0.001** | 0.82 (0.77-0.87) | | 0.89 (0.82-0.97) | | 0.786 | |
| <150 min/wk MVPA and TyG-WHtR Q 1 | 0.48 (0.44-0.51) | 0.72 (0.65-0.80) | | **<0.001** | 0.62 (0.58-0.67) | | 0.81 (0.72-0.90) | | 0.175 | |
| 150-299min/wk MVPA and TyG-WHtR Q 3 | 0.90 (0.84-0.95) | 0.90 (0.82-0.98) | | 0.407 | 0.87 (0.80-0.93) | | 0.91 (0.83-1.00) | | 0.193 | |
| 150-299min/wk MVPA and TyG-WHtR Q 2 | 0.68 (0.64-0.73) | 0.79 (0.72-0.88) | | **<0.001** | 0.64 (0.59-0.70) | | 0.72 (0.65-0.80) | | 0.351 | |
| 150-299min/wk MVPA and TyG-WHtR Q 1 | 0.46 (0.42-0.50) | 0.63 (0.54-0.72) | | **<0.001** | 0.55 (0.50-0.61) | | 0.80 (0.70-0.92) | | 0.061 | |
| 300-599min/wk MVPA and TyG-WHtR Q 3 | 0.93 (0.88-0.99) | 0.88 (0.81-0.96) | | 0.922 | 0.83 (0.77-0.89) | | 0.80 (0.73-0.87) | | 0.641 | |
| 300-599min/wk MVPA and TyG-WHtR Q 2 | 0.68 (0.63-0.72) | 0.78 (0.71-0.86) | | **<0.001** | 0.68 (0.63-0.74) | | 0.74 (0.67-0.82) | | 0.476 | |
| 300-599min/wk MVPA and TyG-WHtR Q 1 | 0.43 (0.39-0.47) | 0.64 (0.56-0.73) | | **<0.001** | 0.53 (0.48-0.58) | | 0.71 (0.62-0.81) | | 0.165 | |
| ≥600min/wk MVPA and TyG-WHtR Q 3 | 1.00 (0.95-1.06) | 0.93 (0.87-1.01) | | 0.119 | 0.87 (0.81-0.93) | | 0.87 (0.81-0.94) | | 0.634 | |
| ≥600min/wk MVPA and TyG-WHtR Q 2 | 0.75 (0.71-0.80) | 0.74 (0.68-0.80) | | **0.048** | 0.71 (0.66-0.76) | | 0.68 (0.62-0.75) | | 0.495 | |
| ≥600min/wk MVPA and TyG-WHtR Q 1 | 0.50 (0.46-0.54) | 0.64 (0.57-0.71) | | **<0.001** | 0.59 (0.54-0.64) | | 0.70 (0.63-0.78) | | 0.506 | |

MVPA, moderate to vigorous physical activity; TyG-WHtR, triglyceride glucose-waist height ratio; CVD, cardiovascular disease; HR, hazard ratio; CI, confidence interval.

The P value for interaction was obtained including the product term of age and the combined variable of MVPA and TyG-WHtR in the multivariable-adjusted model.

The multivariable-adjusted model was adjusted for sex, race, education level, Townsend deprivation index, smoking status, drinking status, parental history of CVD, self-reported use of antihypertensive drugs, self-reported use of lipid-lowering drugs and self-reported use of insulin.

| **Table S7. Associations of TyG-WHtR index with incident CVD and all-cause mortality by MVPA in sex subgroups** | | | | | | | | |
| --- | --- | --- | --- | --- | --- | --- | --- | --- |
|  | **CVD** | | | | **All-cause mortality** | | | |
|  | **Male(n=137193)** | | **Female(n=162735)** | | **Male(n=137193)** | | **Female(n=162735)** | |
|  | **Adjusted HR (95% CI)** | ***P* for interaction** | **Adjusted HR (95% CI)** | ***P* for interaction** | **Adjusted HR (95% CI)** | ***P* for interaction** | **Adjusted HR (95% CI)** | ***P* for interaction** |
| <150min/wk |  |  |  |  |  |  |  |  |
| TyG-WHtR Q 1 | 1.00 (Reference) |  | 1.00 (Reference) |  | 1.00 (Reference) |  | 1.00 (Reference) |  |
| TyG-WHtR Q 2 | 1.30 (1.12-1.51) |  | 1.32 (1.23-1.41) |  | 0.92 (0.79-1.06) |  | 1.18 (1.10-1.27) |  |
| TyG-WHtR Q 3 | 1.68 (1.46-1.95) |  | 1.70 (1.57-1.84) |  | 1.02 (0.89-1.17) |  | 1.52 (1.39-1.65) |  |
| 150-299min/wk |  |  |  |  |  |  |  |  |
| TyG-WHtR Q 1 | 1.00 (Reference) | 0.533 | 1.00 (Reference) | 0.159 | 1.00 (Reference) | 0.595 | 1.00 (Reference) | 0.111 |
| TyG-WHtR Q 2 | 1.53 (1.24-1.88) | 0.125 | 1.19 (1.07-1.33) | 0.605 | 0.85 (0.69-1.05) | 0.029 | 0.98 (0.87-1.10) | 0.655 |
| TyG-WHtR Q 3 | 1.85 (1.51-2.27) |  | 1.48 (1.30-1.69) |  | 1.08 (0.89-1.32) |  | 1.28 (1.11-1.48) |  |
| 300-599min/wk |  |  |  |  |  |  |  |  |
| TyG-WHtR Q 1 | 1.00 (Reference) | 0.781 | 1.00 (Reference) | 0.119 | 1.00 (Reference) | 0.181 | 1.00 (Reference) | 0.014 |
| TyG-WHtR Q 2 | 1.31 (1.11-1.54) | 0.644 | 1.35 (1.22-1.49) | 0.026 | 1.04 (0.86-1.24) | 0.558 | 1.13 (1.02-1.26) | 0.041 |
| TyG-WHtR Q 3 | 1.78 (1.52-2.09) |  | 1.49 (1.31-1.69) |  | 1.21 (1.01-1.44) |  | 1.24 (1.07-1.43) |  |
| ≥600min/wk |  |  |  |  |  |  |  |  |
| TyG-WHtR Q 1 | 1.00 (Reference) | 0.430 | 1.00 (Reference) | 0.033 | 1.00 (Reference) | 0.075 | 1.00 (Reference) | 0.011 |
| TyG-WHtR Q 2 | 1.25 (1.10-1.42) | 0.550 | 1.24 (1.13-1.36) | 0.334 | 1.05 (0.90-1.21) | 0.275 | 0.98 (0.89-1.08) | 0.796 |
| TyG-WHtR Q 3 | 1.61 (1.43-1.82) |  | 1.54 (1.37-1.74) |  | 1.25 (1.09-1.44) |  | 1.23 (1.08-1.40) |  |

MVPA, moderate to vigorous physical activity; TyG-WHtR, triglyceride glucose-waist height ratio; CVD, cardiovascular disease; HR, hazard ratio; CI, confidence interval.

The P value for interaction was obtained including the product term of TyG-WHtR index (tertiles) and MVPA (four groups) in the multivariable-adjusted model, among which TyG-WHtR Q 3 and <150min/wk MVPA was set as the reference group.

The multivariable-adjusted model was adjusted for age, race, education level, Townsend deprivation index, smoking status, drinking status, parental history of CVD, self-reported use of antihypertensive drugs, self-reported use of lipid-lowering drugs and self-reported use of insulin.

| **Table S8. Joint associations of MVPA and TyG-WHtR with incident CVD and all-cause mortality in sex subgroups** | | | | | | | | | | |  |
| --- | --- | --- | --- | --- | --- | --- | --- | --- | --- | --- | --- |
|  | **CVD** | | | | | **All-cause mortality** | | | | |  |
|  | **Adjusted HR (95% CI)** | | ***P* for interaction** | | | **Adjusted HR (95% CI)** | | | ***P* for interaction** | |  |
|  | **Male(n=137193)** | **Female(n=162735)** | |  | **Male(n=137193)** | | **Female(n=162735)** | | |  | |
| <150 min/wk MVPA and TyG-WHtR Q 3 | 1.00 (Reference) | 1.00 (Reference) | |  | 1.00 (Reference) | | | 1.00 (Reference) | |  | |
| <150 min/wk MVPA and TyG-WHtR Q 2 | 0.76 (0.72-0.81) | 0.77 (0.71-0.83) | | 0.379 | 0.89 (0.84-0.95) | | | 0.78 (0.72-0.85) | | **0.020** | |
| <150 min/wk MVPA and TyG-WHtR Q 1 | 0.59 (0.51-0.68) | 0.59 (0.54-0.63) | | 0.229 | 0.97 (0.85-1.12) | | | 0.67 (0.61-0.72) | | **<0.001** | |
| 150-299min/wk MVPA and TyG-WHtR Q 3 | 0.90 (0.85-0.95) | 0.85 (0.76-0.96) | | 0.400 | 0.90 (0.84-0.96) | | | 0.81 (0.71-0.93) | | 0.159 | |
| 150-299min/wk MVPA and TyG-WHtR Q 2 | 0.74 (0.69-0.80) | 0.68 (0.62-0.75) | | **0.027** | 0.70 (0.64-0.77) | | | 0.61 (0.55-0.68) | | 0.064 | |
| 150-299min/wk MVPA and TyG-WHtR Q 1 | 0.49 (0.40-0.59) | 0.56 (0.51-0.61) | | 0.812 | 0.82 (0.68-0.99) | | | 0.62 (0.56-0.68) | | **0.018** | |
| 300-599min/wk MVPA and TyG-WHtR Q 3 | 0.93 (0.88-0.97) | 0.80 (0.71-0.90) | | **0.023** | 0.83 (0.78-0.88) | | | 0.71 (0.62-0.81) | | **0.035** | |
| 300-599min/wk MVPA and TyG-WHtR Q 2 | 0.69 (0.64-0.74) | 0.72 (0.66-0.79) | | 0.900 | 0.71 (0.66-0.77) | | | 0.66 (0.60-0.73) | | 0.234 | |
| 300-599min/wk MVPA and TyG-WHtR Q 1 | 0.53 (0.45-0.61) | 0.52 (0.48-0.58) | | 0.214 | 0.69 (0.58-0.82) | | | 0.58 (0.53-0.64) | | 0.134 | |
| ≥600min/wk MVPA and TyG-WHtR Q 3 | 0.98 (0.93-1.03) | 0.81 (0.73-0.91) | | **0.004** | 0.86 (0.81-0.91) | | | 0.75 (0.66-0.85) | | **0.029** | |
| ≥600min/wk MVPA and TyG-WHtR Q 2 | 0.77 (0.72-0.81) | 0.67 (0.61-0.73) | | **0.001** | 0.73 (0.68-0.78) | | | 0.60 (0.54-0.66) | | **0.001** | |
| ≥600min/wk MVPA and TyG-WHtR Q 1 | 0.62 (0.55-0.70) | 0.55 (0.51-0.60) | | **0.005** | 0.70 (0.61-0.80) | | | 0.61 (0.55-0.66) | | 0.117 | |

MVPA, moderate to vigorous physical activity; TyG-WHtR, triglyceride glucose-waist height ratio; CVD, cardiovascular disease; HR, hazard ratio; CI, confidence interval.

The P value for interaction was obtained including the product term of sex and the combined variable of MVPA and TyG-WHtR in the multivariable-adjusted model.

The multivariable-adjusted model was adjusted for age, race, education level, Townsend deprivation index, smoking status, drinking status, parental history of CVD, self-reported use of antihypertensive drugs, self-reported use of lipid-lowering drugs and self-reported use of insulin.

| **Table S9. Associations of TyG-WHtR index with incident CVD and all-cause mortality by MVPA in race subgroups** | | | | | | | | |
| --- | --- | --- | --- | --- | --- | --- | --- | --- |
|  | **CVD** | | | | **All-cause mortality** | | | |
|  | **White(n=286914)** | | **Non-white(n=13014)** | | **White(n=286914)** | | **Non-white(n=13014)** | |
|  | **Adjusted HR (95% CI)** | ***P* for interaction** | **Adjusted HR (95% CI)** | ***P* for interaction** | **Adjusted HR (95% CI)** | ***P* for interaction** | **Adjusted HR (95% CI)** | ***P* for interaction** |
| <150min/wk |  |  |  |  |  |  |  |  |
| TyG-WHtR Q 1 | 1.00 (Reference) |  | 1.00 (Reference) |  | 1.00 (Reference) |  | 1.00 (Reference) |  |
| TyG-WHtR Q 2 | 1.34 (1.26-1.43) |  | 1.75 (1.27-2.40) |  | 1.14 (1.07-1.22) |  | 1.11 (0.74-1.67) |  |
| TyG-WHtR Q 3 | 1.74 (1.63-1.86) |  | 2.37 (1.71-3.28) |  | 1.32 (1.24-1.42) |  | 1.51 (1.00-2.28) |  |
| 150-299min/wk |  |  |  |  |  |  |  |  |
| TyG-WHtR Q 1 | 1.00 (Reference) | 0.453 | 1.00 (Reference) | 0.407 | 1.00 (Reference) | 0.472 | 1.00 (Reference) | 0.778 |
| TyG-WHtR Q 2 | 1.34 (1.22-1.47) | 0.238 | **1.04 (0.65-1.67)** | 0.504 | 0.97 (0.87-1.07) | 0.030 | 0.53 (0.29-0.94) | 0.038 |
| TyG-WHtR Q 3 | 1.64 (1.49-1.82) |  | 1.47 (0.91-2.35) |  | 1.22 (1.10-1.36) |  | 1.16 (0.68-2.00) |  |
| 300-599min/wk |  |  |  |  |  |  |  |  |
| TyG-WHtR Q 1 | 1.00 (Reference) | 0.858 | 1.00 (Reference) | 0.979 | 1.00 (Reference) | 0.326 | 1.00 (Reference) | 0.634 |
| TyG-WHtR Q 2 | 1.37 (1.26-1.49) | 0.616 | 1.67 (1.06-2.63) | 0.847 | 1.08 (0.99-1.19) | 0.777 | 1.23 (0.71-2.12) | 0.442 |
| TyG-WHtR Q 3 | 1.74 (1.59-1.91) |  | 2.31 (1.44-3.72) |  | 1.23 (1.12-1.37) |  | 1.40 (0.77-2.55) |  |
| ≥600min/wk |  |  |  |  |  |  |  |  |
| TyG-WHtR Q 1 | 1.00 (Reference) | 0.627 | 1.00 (Reference) | 0.384 | 1.00 (Reference) | 0.364 | 1.00 (Reference) | 0.918 |
| TyG-WHtR Q 2 | 1.29 (1.19-1.38) | 0.809 | **0.92 (0.61-1.40)** | 0.036 | 1.01 (0.93-1.09) | 0.133 | 0.99 (0.59-1.65) | 0.899 |
| TyG-WHtR Q 3 | 1.63 (1.50-1.76) |  | 1.85 (1.22-2.81) |  | 1.23 (1.13-1.35) |  | 1.09 (0.62-1.89) |  |

MVPA, moderate to vigorous physical activity; TyG-WHtR, triglyceride glucose-waist height ratio; CVD, cardiovascular disease; HR, hazard ratio; CI, confidence interval.

The P value for interaction was obtained including the product term of TyG-WHtR index (tertiles) and MVPA (four groups) in the multivariable-adjusted model, among which TyG-WHtR Q 3 and <150min/wk MVPA was set as the reference group.

The multivariable-adjusted model was adjusted for age, sex, education level, Townsend deprivation index, smoking status, drinking status, parental history of CVD, self-reported use of antihypertensive drugs, self-reported use of lipid-lowering drugs and self-reported use of insulin.

| **Table S10. Joint associations of MVPA and TyG-WHtR with incident CVD and all-cause mortality in race subgroups** | | | | | | | | | | | | | | | |
| --- | --- | --- | --- | --- | --- | --- | --- | --- | --- | --- | --- | --- | --- | --- | --- |
|  | **CVD** | | | | | | | | **All-cause mortality** | | | | | | |
|  | **Adjusted HR (95% CI)** | | | ***P* for interaction** | | | | | **Adjusted HR (95% CI)** | | ***P* for interaction** | | | | |
|  | **White(n=286914)** | **Non-white(n=13014)** | | | |  | **White(n=286914)** | | | **Non-white(n=13014)** | | |  | |  |
| <150 min/wk MVPA and TyG-WHtR Q 3 | 1.00 (Reference) | | 1.00 (Reference) | |  | | | 1.00 (Reference) | | 1.00 (Reference) | |  | |  |  |
| <150 min/wk MVPA and TyG-WHtR Q 2 | 0.78 (0.74-0.81) | | 0.76 (0.62-0.94) | | 0.947 | | | 0.87 (0.83-0.91) | | 0.78 (0.58-1.06) | | 0.698 | |  |  |
| <150 min/wk MVPA and TyG-WHtR Q 1 | 0.58 (0.55-0.62) | | 0.45 (0.33-0.61) | | 0.119 | | | 0.77 (0.72-0.82) | | 0.71 (0.49-1.05) | | 0.915 | |  |  |
| 150-299min/wk MVPA and TyG-WHtR Q 3 | 0.90 (0.85-0.94) | | 0.87 (0.69-1.11) | | 0.936 | | | 0.87 (0.82-0.92) | | 1.26 (0.93-1.72) | | 0.018 | |  |  |
| 150-299min/wk MVPA and TyG-WHtR Q 2 | 0.73 (0.69-0.77) | | 0.58 (0.43-0.79) | | 0.180 | | | 0.68 (0.64-0.73) | | 0.54 (0.34-0.85) | | 0.427 | |  |  |
| 150-299min/wk MVPA and TyG-WHtR Q 1 | 0.54 (0.50-0.59) | | 0.49 (0.33-0.72) | | 0.666 | | | 0.70 (0.64-0.75) | | 0.98 (0.64-1.50) | | 0.037 | |  |  |
| 300-599min/wk MVPA and TyG-WHtR Q 3 | 0.90 (0.86-0.95) | | 0.90 (0.72-1.13) | | 0.982 | | | 0.80 (0.76-0.85) | | 0.83 (0.59-1.17) | | 0.768 | |  |  |
| 300-599min/wk MVPA and TyG-WHtR Q 2 | 0.72 (0.68-0.76) | | 0.66 (0.51-0.87) | | 0.706 | | | 0.70 (0.66-0.75) | | 0.80 (0.55-1.16) | | 0.304 | |  |  |
| 300-599min/wk MVPA and TyG-WHtR Q 1 | 0.52 (0.49-0.56) | | 0.41 (0.27-0.61) | | 0.287 | | | 0.65 (0.60-0.70) | | 0.69 (0.43-1.11) | | 0.425 | |  |  |
| ≥600min/wk MVPA and TyG-WHtR Q 3 | 0.95 (0.91-1.00) | | 0.96 (0.76-1.19) | | 0.947 | | | 0.84 (0.80-0.88) | | 0.97 (0.71-1.34) | | 0.309 | |  |  |
| ≥600min/wk MVPA and TyG-WHtR Q 2 | 0.75 (0.71-0.78) | | 0.49 (0.36-0.65) | | 0.004 | | | 0.68 (0.65-0.72) | | 0.79 (0.55-1.13) | | 0.350 | |  |  |
| ≥600min/wk MVPA and TyG-WHtR Q 1 | 0.57 (0.53-0.61) | | 0.53 (0.38-0.74) | | 0.723 | | | 0.67 (0.63-0.72) | | 0.72 (0.46-1.11) | | 0.490 | |  |  |

MVPA, moderate to vigorous physical activity; TyG-WHtR, triglyceride glucose-waist height ratio; CVD, cardiovascular disease; HR, hazard ratio; CI, confidence interval.

The P value for interaction was obtained including the product term of race and the combined variable of MVPA and TyG-WHtR in the multivariable-adjusted model.

The multivariable-adjusted model was adjusted for age, sex, education level, Townsend deprivation index, smoking status, drinking status, parental history of CVD, self-reported use of antihypertensive drugs, self-reported use of lipid-lowering drugs and self-reported use of insulin.

| **Table S11. Associations of TyG-WHtR index with incident CVD and all-cause mortality by MVPA in education level subgroups** | | | | | | | | |
| --- | --- | --- | --- | --- | --- | --- | --- | --- |
|  | **CVD** | | | | **All-cause mortality** | | | |
|  | **Low(n=144308)** | | **High(n=155620)** | | **Low(n=144308)** | | **High(n=155620)** | |
|  | **Adjusted HR (95% CI)** | ***P* for interaction** | **Adjusted HR (95% CI)** | ***P* for interaction** | **Adjusted HR (95% CI)** | ***P* for interaction** | **Adjusted HR (95% CI)** | ***P* for interaction** |
| <150min/wk |  |  |  |  |  |  |  |  |
| TyG-WHtR Q 1 | 1.00 (Reference) |  | 1.00 (Reference) |  | 1.00 (Reference) |  | 1.00 (Reference) |  |
| TyG-WHtR Q 2 | 1.32 (1.21-1.43) |  | 1.40 (1.28-1.53) |  | 1.15 (1.05-1.25) |  | 1.14 (1.04-1.25) |  |
| TyG-WHtR Q 3 | 1.70 (1.56-1.86) |  | 1.83 (1.66-2.01) |  | 1.34 (1.22-1.47) |  | 1.33 (1.20-1.47) |  |
| 150-299min/wk |  |  |  |  |  |  |  |  |
| TyG-WHtR Q 1 | 1.00 (Reference) | 0.932 | 1.00 (Reference) | 0.242 | 1.00 (Reference) | 0.514 | 1.00 (Reference) | 0.763 |
| TyG-WHtR Q 2 | 1.36 (1.19-1.55) | 0.345 | 1.28 (1.13-1.46) | 0.598 | 0.91 (0.79-1.04) | 0.031 | 1.01 (0.87-1.16) | 0.155 |
| TyG-WHtR Q 3 | 1.65 (1.44-1.90) |  | 1.59 (1.39-1.83) |  | 1.16 (1.00-1.34) |  | 1.28 (1.10-1.49) |  |
| 300-599min/wk |  |  |  |  |  |  |  |  |
| TyG-WHtR Q 1 | 1.00 (Reference) | 0.661 | 1.00 (Reference) | 0.769 | 1.00 (Reference) | 0.146 | 1.00 (Reference) | 0.934 |
| TyG-WHtR Q 2 | 1.33 (1.18-1.49) | 0.838 | 1.43 (1.27-1.61) | 0.381 | 1.06 (0.93-1.20) | 0.509 | 1.13 (0.99-1.29) | 0.907 |
| TyG-WHtR Q 3 | 1.72 (1.52-1.95) |  | 1.78 (1.56-2.02) |  | 1.17 (1.02-1.35) |  | 1.33 (1.16-1.54) |  |
| ≥600min/wk |  |  |  |  |  |  |  |  |
| TyG-WHtR Q 1 | 1.00 (Reference) | 0.575 | 1.00 (Reference) | 0.161 | 1.00 (Reference) | 0.644 | 1.00 (Reference) | 0.420 |
| TyG-WHtR Q 2 | 1.29 (1.17-1.42) | 0.641 | 1.26 (1.13-1.40) | 0.786 | 1.04 (0.93-1.16) | 0.358 | 0.97 (0.86-1.10) | 0.241 |
| TyG-WHtR Q 3 | 1.65 (1.49-1.83) |  | 1.59 (1.42-1.79) |  | 1.25 (1.12-1.40) |  | 1.20 (1.05-1.37) |  |

MVPA, moderate to vigorous physical activity; TyG-WHtR, triglyceride glucose-waist height ratio; CVD, cardiovascular disease; HR, hazard ratio; CI, confidence interval.

The P value for interaction was obtained including the product term of TyG-WHtR index (tertiles) and MVPA (four groups) in the multivariable-adjusted model, among which TyG-WHtR Q 3 and <150min/wk MVPA was set as the reference group.

The multivariable-adjusted model was adjusted for age, sex, race, Townsend deprivation index, smoking status, drinking status, parental history of CVD, self-reported use of antihypertensive drugs, self-reported use of lipid-lowering drugs and self-reported use of insulin.

| **Table S12. Joint associations of MVPA and TyG-WHtR with incident CVD and all-cause mortality in education level subgroups** | | | | | | | | | | | | | | |
| --- | --- | --- | --- | --- | --- | --- | --- | --- | --- | --- | --- | --- | --- | --- |
|  | **CVD** | | | | | | | **All-cause mortality** | | | | | | |
|  | **Adjusted HR (95% CI)** | | ***P* for interaction** | | | | | **Adjusted HR (95% CI)** | | ***P* for interaction** | | | | |
|  | **Low(n=144308)** | **High(n=155620)** | | |  | **Low(n=144308)** | | | **High(n=155620)** | | |  | |  |
| <150 min/wk MVPA and TyG-WHtR Q 3 | 1.00 (Reference) | 1.00 (Reference) | |  | | | 1.00 (Reference) | | 1.00 (Reference) | |  | |  |  |
| <150 min/wk MVPA and TyG-WHtR Q 2 | 0.79 (0.74-0.83) | 0.77 (0.72-0.82) | | 0.704 | | | 0.87 (0.81-0.93) | | 0.86 (0.80-0.93) | | 0.394 | |  |  |
| <150 min/wk MVPA and TyG-WHtR Q 1 | 0.60 (0.56-0.65) | 0.56 (0.51-0.61) | | 0.172 | | | 0.77 (0.71-0.84) | | 0.76 (0.70-0.83) | | 0.118 | |  |  |
| 150-299min/wk MVPA and TyG-WHtR Q 3 | 0.88 (0.82-0.94) | 0.91 (0.85-0.98) | | 0.375 | | | 0.88 (0.81-0.95) | | 0.89 (0.82-0.97) | | 0.669 | |  |  |
| 150-299min/wk MVPA and TyG-WHtR Q 2 | 0.73 (0.67-0.79) | 0.73 (0.67-0.79) | | 0.913 | | | 0.66 (0.60-0.73) | | 0.70 (0.63-0.77) | | 0.140 | |  |  |
| 150-299min/wk MVPA and TyG-WHtR Q 1 | 0.53 (0.48-0.59) | 0.56 (0.50-0.62) | | 0.542 | | | 0.71 (0.63-0.79) | | 0.70 (0.62-0.78) | | 0.259 | |  |  |
| 300-599min/wk MVPA and TyG-WHtR Q 3 | 0.90 (0.85-0.96) | 0.91 (0.84-0.97) | | 0.837 | | | 0.78 (0.73-0.84) | | 0.83 (0.76-0.91) | | 0.266 | |  |  |
| 300-599min/wk MVPA and TyG-WHtR Q 2 | 0.70 (0.65-0.76) | 0.73 (0.68-0.79) | | 0.324 | | | 0.71 (0.65-0.77) | | 0.71 (0.65-0.78) | | 0.405 | |  |  |
| 300-599min/wk MVPA and TyG-WHtR Q 1 | 0.53 (0.48-0.58) | 0.52 (0.47-0.57) | | 0.803 | | | 0.67 (0.60-0.74) | | 0.63 (0.57-0.70) | | 0.552 | |  |  |
| ≥600min/wk MVPA and TyG-WHtR Q 3 | 0.94 (0.89-0.99) | 0.97 (0.91-1.04) | | 0.371 | | | 0.82 (0.77-0.88) | | 0.87 (0.80-0.95) | | 0.344 | |  |  |
| ≥600min/wk MVPA and TyG-WHtR Q 2 | 0.72 (0.68-0.77) | 0.76 (0.71-0.82) | | 0.212 | | | 0.68 (0.63-0.73) | | 0.69 (0.63-0.76) | | 0.348 | |  |  |
| ≥600min/wk MVPA and TyG-WHtR Q 1 | 0.55 (0.50-0.60) | 0.60 (0.54-0.65) | | 0.138 | | | 0.65 (0.59-0.71) | | 0.70 (0.63-0.77) | | 0.013 | |  |  |

MVPA, moderate to vigorous physical activity; TyG-WHtR, triglyceride glucose-waist height ratio; CVD, cardiovascular disease; HR, hazard ratio; CI, confidence interval.

The P value for interaction was obtained including the product term of education level and the combined variable of MVPA and TyG-WHtR in the multivariable-adjusted model.

The multivariable-adjusted model was adjusted for age, sex, race, Townsend deprivation index, smoking status, drinking status, parental history of CVD, self-reported use of antihypertensive drugs, self-reported use of lipid-lowering drugs and self-reported use of insulin.

| **Table S13. Independent associations of MVPA with incident CVD and all-cause mortality after excluding participants who had outcomes within the first two years of follow-up** | | | | |
| --- | --- | --- | --- | --- |
|  | **CVD** | | **All-cause mortality** | |
|  | **Unadjusted HR (95% CI)** | **Adjusted HR (95% CI)** | **Unadjusted HR (95% CI)** | **Adjusted HR (95% CI)** |
| <150 min/wk | 1.00 (Reference) | 1.00 (Reference) | 1.00 (Reference) | 1.00 (Reference) |
| 150-299 min/wk | 0.86 (0.83-0.90) | 0.90 (0.87-0.93) | 0.82 (0.79-0.85) | 0.85 (0.82-0.89) |
| 300-599 min/wk | 0.86 (0.83-0.89) | 0.88 (0.85-0.91) | 0.80 (0.77-0.83) | 0.82 (0.79-0.85) |
| ≥600 min/wk | 1.00 (0.97-1.03) | 0.93 (0.90-0.96) | 0.90 (0.87-0.93) | 0.82 (0.79-0.85) |
|  | *P* trend=0.396 | *P* trend<0.001 | *P* trend<0.001 | *P* trend<0.001 |

MVPA, moderate to vigorous physical activity; CVD, cardiovascular disease; HR, hazard ratio; CI, confidence interval.

The multivariable-adjusted model was adjusted for age, sex, race, education level, Townsend deprivation index, smoking status, drinking status, parental history of CVD, self-reported use of antihypertensive drugs, self-reported use of lipid-lowering drugs and self-reported use of insulin.

| **Table S14. Independent associations of TyG-WHtR index with incident CVD and all-cause mortality** **after excluding participants who had outcomes within the first two years of follow-up** | | | | |
| --- | --- | --- | --- | --- |
|  | **CVD** | | **All-cause mortality** | |
|  | **Unadjusted HR (95% CI)** | **Adjusted HR (95% CI)** | **Unadjusted HR (95% CI)** | **Adjusted HR (95% CI)** |
| TyG-WHtR (per unit) | 1.47 (1.46-1.48) | 1.24 (1.23-1.26) | 1.38 (1.37-1.40) | 1.15 (1.13-1.17) |
| TyG-WHtR (tertiles) |  |  |  |  |
| Q 1 | 1.00 (Reference) | 1.00 (Reference) | 1.00 (Reference) | 1.00 (Reference) |
| Q 2 | 1.90 (1.83-1.98) | 1.33 (1.28-1.38) | 1.53 (1.47-1.59) | 1.07 (1.03-1.11) |
| Q 3 | 3.10 (2.99-3.21) | 1.69 (1.62-1.76) | 2.30 (2.22-2.38) | 1.28 (1.23-1.34) |
|  | *P* trend<0.001 | *P* trend<0.001 | *P* trend<0.001 | *P* trend<0.001 |

TyG-WHtR, triglyceride glucose-waist height ratio; CVD, cardiovascular disease; HR, hazard ratio; CI, confidence interval.

The multivariable-adjusted model was adjusted for age, sex, race, education level, Townsend deprivation index, smoking status, drinking status, MVPA, parental history of CVD, self-reported use of antihypertensive drugs, self-reported use of lipid-lowering drugs and self-reported use of insulin.

| **Table S15. Associations of TyG-WHtR index with incident CVD and all-cause mortality by MVPA after excluding participants who had outcomes within the first two years of follow-up** | | | | | | |
| --- | --- | --- | --- | --- | --- | --- |
|  | **CVD** | | | **All-cause mortality** | | |
|  | **Unadjusted**  **HR (95% CI)** | **Adjusted**  **HR (95% CI)** | ***P* for interaction** | **Unadjusted**  **HR (95% CI)** | **Adjusted**  **HR (95% CI)** | ***P* for interaction** |
| <150min/wk |  |  |  |  |  |  |
| TyG-WHtR Q 1 | 1.00 (Reference) | 1.00 (Reference) |  | 1.00 (Reference) | 1.00 (Reference) |  |
| TyG-WHtR Q 2 | 1.91 (1.80-2.03) | 1.36 (1.28-1.45) |  | 1.60 (1.51-1.71) | 1.14 (1.06-1.21) |  |
| TyG-WHtR Q 3 | 3.19 (3.02-3.38) | 1.74 (1.63-1.87) |  | 2.39 (2.26-2.54) | 1.34 (1.25-1.44) |  |
| 150-299min/wk |  |  |  |  |  |  |
| TyG-WHtR Q 1 | 1.00 (Reference) | 1.00 (Reference) | 0.555 | 1.00 (Reference) | 1.00 (Reference) | 0.557 |
| TyG-WHtR Q 2 | 1.91 (1.75-2.09) | 1.31 (1.19-1.44) | 0.662 | 1.39 (1.27-1.53) | 0.95 (0.85-1.05)* | 0.010 |
| TyG-WHtR Q 3 | 3.05 (2.80-3.32) | 1.62 (1.47-1.80) |  | 2.28 (2.08-2.49) | 1.23 (1.10-1.37) |  |
| 300-599min/wk |  |  |  |  |  |  |
| TyG-WHtR Q 1 | 1.00 (Reference) | 1.00 (Reference) | 0.661 | 1.00 (Reference) | 1.00 (Reference) | 0.183 |
| TyG-WHtR Q 2 | 1.93 (1.78-2.09) | 1.32 (1.21-1.44) | 0.899 | 1.59 (1.46-1.73) | 1.09 (1.00-1.20)* | 0.315 |
| TyG-WHtR Q 3 | 3.12 (2.89-3.37) | 1.67 (1.52-1.83) |  | 2.23 (2.05-2.43) | 1.22 (1.10-1.35) |  |
| ≥600min/wk |  |  |  |  |  |  |
| TyG-WHtR Q 1 | 1.00 (Reference) | 1.00 (Reference) | 0.566 | 1.00 (Reference) | 1.00 (Reference) | 0.244 |
| TyG-WHtR Q 2 | 1.86 (1.74-2.00) | 1.28 (1.19-1.38) | 0.989 | 1.44 (1.33-1.56) | 1.01 (0.93-1.09)* | 0.381 |
| TyG-WHtR Q 3 | 2.93 (2.74-3.14) | 1.60 (1.48-1.74) |  | 2.12 (1.97-2.28) | 1.21 (1.11-1.32) |  |

MVPA, moderate to vigorous physical activity; TyG-WHtR, triglyceride glucose-waist height ratio; CVD, cardiovascular disease; HR, hazard ratio; CI, confidence interval.

The P value for interaction was obtained including the product term of TyG-WHtR index (tertiles) and MVPA (four groups) in the multivariable-adjusted model, among which TyG-WHtR Q 3 and <150min/wk MVPA was set as the reference group.

The multivariable-adjusted model was adjusted for age, sex, race, education level, Townsend deprivation index, smoking status, drinking status, parental history of CVD, self-reported use of antihypertensive drugs, self-reported use of lipid-lowering drugs and self-reported use of insulin. *Value indicates no statistical significance.

| **Table S16. Joint associations of MVPA and TyG-WHtR with incident CVD and all-cause mortality after excluding participants who had outcomes within the first two years of follow-up** | | | | |
| --- | --- | --- | --- | --- |
|  | **CVD** | | **All-cause mortality** | |
|  | **Unadjusted HR (95% CI)** | **Adjusted HR (95% CI)** | **Unadjusted HR (95% CI)** | **Adjusted HR (95% CI)** |
| <150 min/wk MVPA and TyG-WHtR Q 3 | 1.00 (Reference) | 1.00 (Reference) | 1.00 (Reference) | 1.00 (Reference) |
| <150 min/wk MVPA and TyG-WHtR Q 2 | 0.60 (0.57-0.62) | 0.79 (0.76-0.83) | 0.67 (0.64-0.70) | 0.86 (0.82-0.90) |
| <150 min/wk MVPA and TyG-WHtR Q 1 | 0.31 (0.30-0.33) | 0.59 (0.55-0.63) | 0.42 (0.39-0.44) | 0.77 (0.72-0.82) |
| 150-299min/wk MVPA and TyG-WHtR Q 3 | 0.89 (0.85-0.94) | 0.91 (0.86-0.96) | 0.87 (0.82-0.93) | 0.89 (0.84-0.94) |
| 150-299min/wk MVPA and TyG-WHtR Q 2 | 0.56 (0.53-0.60) | 0.73 (0.69-0.78) | 0.54 (0.50-0.57) | 0.67 (0.63-0.72) |
| 150-299min/wk MVPA and TyG-WHtR Q 1 | 0.29 (0.27-0.32) | 0.55 (0.51-0.60) | 0.38 (0.36-0.42) | 0.70 (0.65-0.76) |
| 300-599min/wk MVPA and TyG-WHtR Q 3 | 0.91 (0.87-0.96) | 0.90 (0.86-0.95) | 0.82 (0.77-0.87) | 0.81 (0.76-0.85) |
| 300-599min/wk MVPA and TyG-WHtR Q 2 | 0.56 (0.53-0.59) | 0.72 (0.68-0.76) | 0.58 (0.55-0.62) | 0.72 (0.68-0.77) |
| 300-599min/wk MVPA and TyG-WHtR Q 1 | 0.29 (0.27-0.31) | 0.54 (0.51-0.59) | 0.37 (0.34-0.39) | 0.66 (0.61-0.71) |
| ≥600min/wk MVPA and TyG-WHtR Q 3 | 1.02 (0.98-1.07) | 0.96 (0.92-1.00) | 0.92 (0.87-0.97) | 0.84 (0.79-0.88) |
| ≥600min/wk MVPA and TyG-WHtR Q 2 | 0.65 (0.62-0.69) | 0.76 (0.72-0.80) | 0.62 (0.59-0.66) | 0.69 (0.65-0.74) |
| ≥600min/wk MVPA and TyG-WHtR Q 1 | 0.35 (0.33-0.37) | 0.58 (0.54-0.62) | 0.43 (0.41-0.46) | 0.68 (0.63-0.73) |

MVPA, moderate to vigorous physical activity; TyG-WHtR, triglyceride glucose-waist height ratio; CVD, cardiovascular disease; HR, hazard ratio; CI, confidence interval.

The multivariable-adjusted model was adjusted for age, sex, race, education level, Townsend deprivation index, smoking status, drinking status, parental history of CVD, self-reported use of antihypertensive drugs, self-reported use of lipid-lowering drugs and self-reported use of insulin.

| **Table S17. Independent associations of MVPA with incident CVD and all-cause mortality after imputing all missing covariates** | | | | |
| --- | --- | --- | --- | --- |
|  | **CVD** | | **All-cause mortality** | |
|  | **Unadjusted HR (95% CI)** | **Adjusted HR (95% CI)** | **Unadjusted HR (95% CI)** | **Adjusted HR (95% CI)** |
| <150 min/wk | 1.00 (Reference) | 1.00 (Reference) | 1.00 (Reference) | 1.00 (Reference) |
| 150-299 min/wk | 0.85 (0.82-0.88) | 0.89 (0.86-0.92) | 0.81 (0.78-0.84) | 0.84 (0.81-0.87) |
| 300-599 min/wk | 0.85 (0.82-0.88) | 0.88 (0.85-0.91) | 0.78 (0.75-0.80) | 0.80 (0.77-0.83) |
| ≥600 min/wk | 0.98 (0.95-1.01) | 0.92 (0.89-0.94) | 0.89 (0.86-0.92) | 0.81 (0.79-0.84) |
|  | *P* trend=0.855 | *P* trend<0.001 | *P* trend<0.001 | *P* trend<0.001 |

MVPA, moderate to vigorous physical activity; CVD, cardiovascular disease; HR, hazard ratio; CI, confidence interval.

The multivariable-adjusted model was adjusted for age, sex, race, education level, Townsend deprivation index, smoking status, drinking status, parental history of CVD, self-reported use of antihypertensive drugs, self-reported use of lipid-lowering drugs and self-reported use of insulin.

| **Table S18. Independent associations of TyG-WHtR index with incident CVD and all-cause mortality after imputing all missing covariates** | | | | |
| --- | --- | --- | --- | --- |
|  | **CVD** | | **All-cause mortality** | |
|  | **Unadjusted HR (95% CI)** | **Adjusted HR (95% CI)** | **Unadjusted HR (95% CI)** | **Adjusted HR (95% CI)** |
| TyG-WHtR (per unit) | 1.48 (1.47-1.49) | 1.25 (1.24-1.27) | 1.39 (1.38-1.41) | 1.16 (1.14-1.17) |
| TyG-WHtR (tertiles) |  |  |  |  |
| Q 1 | 1.00 (Reference) | 1.00 (Reference) | 1.00 (Reference) | 1.00 (Reference) |
| Q 2 | 1.92 (1.86-1.99) | 1.33 (1.29-1.38) | 1.55 (1.50-1.61) | 1.08 (1.04-1.12) |
| Q 3 | 3.18 (3.08-3.28) | 1.70 (1.63-1.76) | 2.35 (2.28-2.43) | 1.30 (1.25-1.35) |
|  | *P* trend<0.001 | *P* trend<0.001 | *P* trend<0.001 | *P* trend<0.001 |

TyG-WHtR, triglyceride glucose-waist height ratio; CVD, cardiovascular disease; HR, hazard ratio; CI, confidence interval.

The multivariable-adjusted model was adjusted for age, sex, race, education level, Townsend deprivation index, smoking status, drinking status, MVPA, parental history of CVD, self-reported use of antihypertensive drugs, self-reported use of lipid-lowering drugs and self-reported use of insulin.

| **Table S19. Associations of TyG-WHtR index with incident CVD and all-cause mortality by MVPA after imputing all missing covariates** | | | | | | |
| --- | --- | --- | --- | --- | --- | --- |
|  | **CVD** | | | **All-cause mortality** | | |
|  | **Unadjusted**  **HR (95% CI)** | **Adjusted**  **HR (95% CI)** | ***P* for interaction** | **Unadjusted**  **HR (95% CI)** | **Adjusted**  **HR (95% CI)** | ***P* for interaction** |
| <150min/wk |  |  |  |  |  |  |
| TyG-WHtR Q 1 | 1.00 (Reference) | 1.00 (Reference) |  | 1.00 (Reference) | 1.00 (Reference) |  |
| TyG-WHtR Q 2 | 1.91 (1.81-2.02) | 1.35 (1.27-1.43) |  | 1.64 (1.55-1.74) | 1.15 (1.09-1.23) |  |
| TyG-WHtR Q 3 | 3.25 (3.09-3.42) | 1.72 (1.62-1.83) |  | 2.46 (2.33-2.59) | 1.34 (1.26-1.43) |  |
| 150-299min/wk |  |  |  |  |  |  |
| TyG-WHtR Q 1 | 1.00 (Reference) | 1.00 (Reference) | 0.674 | 1.00 (Reference) | 1.00 (Reference) | 0.301 |
| TyG-WHtR Q 2 | 1.91 (1.76-2.08) | 1.30 (1.19-1.42) | 0.913 | 1.42 (1.30-1.55) | 0.96 (0.88-1.06)* | 0.012 |
| TyG-WHtR Q 3 | 3.12 (2.88-3.37) | 1.64 (1.50-1.80) |  | 2.27 (2.10-2.47) | 1.24 (1.12-1.37) |  |
| 300-599min/wk |  |  |  |  |  |  |
| TyG-WHtR Q 1 | 1.00 (Reference) | 1.00 (Reference) | 0.707 | 1.00 (Reference) | 1.00 (Reference) | 0.170 |
| TyG-WHtR Q 2 | 2.03 (1.88-2.19) | 1.39 (1.28-1.50) | 0.609 | 1.61 (1.49-1.75) | 1.10 (1.01-1.20) | 0.479 |
| TyG-WHtR Q 3 | 3.28 (3.06-3.53) | 1.75 (1.60-1.90) |  | 2.30 (2.13-2.48) | 1.24 (1.13-1.36) |  |
| ≥600min/wk |  |  |  |  |  |  |
| TyG-WHtR Q 1 | 1.00 (Reference) | 1.00 (Reference) | 0.455 | 1.00 (Reference) | 1.00 (Reference) | 0.145 |
| TyG-WHtR Q 2 | 1.85 (1.73-1.97) | 1.27 (1.18-1.36) | 0.898 | 1.45 (1.35-1.56) | 1.02 (0.95-1.10)* | 0.229 |
| TyG-WHtR Q 3 | 2.96 (2.78-3.15) | 1.61 (1.49-1.73) |  | 2.17 (2.03-2.32) | 1.24 (1.15-1.35) |  |

MVPA, moderate to vigorous physical activity; TyG-WHtR, triglyceride glucose-waist height ratio; CVD, cardiovascular disease; HR, hazard ratio; CI, confidence interval.

The P value for interaction was obtained including the product term of TyG-WHtR index (tertiles) and MVPA (four groups) in the multivariable-adjusted model, among which TyG-WHtR Q 3 and <150min/wk MVPA was set as the reference group.

The multivariable-adjusted model was adjusted for age, sex, race, education level, Townsend deprivation index, smoking status, drinking status, parental history of CVD, self-reported use of antihypertensive drugs, self-reported use of lipid-lowering drugs and self-reported use of insulin. *Value indicates no statistical significance.

| **Table S20. Joint associations of MVPA and TyG-WHtR with incident CVD and all-cause mortality after imputing all missing covariates** | | | | |
| --- | --- | --- | --- | --- |
|  | **CVD** | | **All-cause mortality** | |
|  | **Unadjusted HR (95% CI)** | **Adjusted HR (95% CI)** | **Unadjusted HR (95% CI)** | **Adjusted HR (95% CI)** |
| <150 min/wk MVPA and TyG-WHtR Q 3 | 1.00 (Reference) | 1.00 (Reference) | 1.00 (Reference) | 1.00 (Reference) |
| <150 min/wk MVPA and TyG-WHtR Q 2 | 0.59 (0.56-0.61) | 0.79 (0.75-0.82) | 0.67 (0.64-0.70) | 0.86 (0.82-0.90) |
| <150 min/wk MVPA and TyG-WHtR Q 1 | 0.31 (0.29-0.32) | 0.59 (0.56-0.62) | 0.41 (0.39-0.43) | 0.75 (0.71-0.80) |
| 150-299min/wk MVPA and TyG-WHtR Q 3 | 0.89 (0.84-0.93) | 0.91 (0.86-0.95) | 0.85 (0.81-0.90) | 0.87 (0.83-0.92) |
| 150-299min/wk MVPA and TyG-WHtR Q 2 | 0.54 (0.52-0.57) | 0.72 (0.68-0.76) | 0.53 (0.50-0.57) | 0.67 (0.63-0.72) |
| 150-299min/wk MVPA and TyG-WHtR Q 1 | 0.28 (0.27-0.31) | 0.54 (0.51-0.59) | 0.38 (0.35-0.40) | 0.69 (0.64-0.75) |
| 300-599min/wk MVPA and TyG-WHtR Q 3 | 0.90 (0.86-0.94) | 0.90 (0.86-0.94) | 0.80 (0.76-0.84) | 0.79 (0.75-0.83) |
| 300-599min/wk MVPA and TyG-WHtR Q 2 | 0.56 (0.53-0.59) | 0.72 (0.69-0.76) | 0.56 (0.53-0.59) | 0.70 (0.66-0.74) |
| 300-599min/wk MVPA and TyG-WHtR Q 1 | 0.28 (0.26-0.29) | 0.52 (0.49-0.56) | 0.35 (0.32-0.37) | 0.63 (0.59-0.68) |
| ≥600min/wk MVPA and TyG-WHtR Q 3 | 1.01 (0.97-1.05) | 0.95 (0.91-0.99) | 0.91 (0.86-0.95) | 0.83 (0.79-0.87) |
| ≥600min/wk MVPA and TyG-WHtR Q 2 | 0.63 (0.60-0.66) | 0.74 (0.71-0.78) | 0.61 (0.58-0.64) | 0.69 (0.65-0.72) |
| ≥600min/wk MVPA and TyG-WHtR Q 1 | 0.34 (0.32-0.36) | 0.58 (0.54-0.61) | 0.42 (0.39-0.45) | 0.67 (0.63-0.71) |

MVPA, moderate to vigorous physical activity; TyG-WHtR, triglyceride glucose-waist height ratio; CVD, cardiovascular disease; HR, hazard ratio; CI, confidence interval.

The multivariable-adjusted model was adjusted for age, sex, race, education level, Townsend deprivation index, smoking status, drinking status, parental history of CVD, self-reported use of antihypertensive drugs, self-reported use of lipid-lowering drugs and self-reported use of insulin.

| **Table S21. Independent associations of MVPA with incident CVD and all-cause mortality after further adjusting sedentary behavior in multivariable-adjusted model** | | | | |
| --- | --- | --- | --- | --- |
|  | **CVD** | | **All-cause mortality** | |
|  | **Unadjusted HR (95% CI)** | **Adjusted HR (95% CI)** | **Unadjusted HR (95% CI)** | **Adjusted HR (95% CI)** |
| <150 min/wk | 1.00 (Reference) | 1.00 (Reference) | 1.00 (Reference) | 1.00 (Reference) |
| 150-299 min/wk | 0.86 (0.83-0.89) | 0.90 (0.87-0.93) | 0.82 (0.78-0.85) | 0.85 (0.82-0.89) |
| 300-599 min/wk | 0.85 (0.82-0.88) | 0.89 (0.86-0.92) | 0.79 (0.76-0.82) | 0.81 (0.78-0.84) |
| ≥600 min/wk | 0.99 (0.96-1.02) | 0.93 (0.90-0.96) | 0.90 (0.87-0.93) | 0.82 (0.79-0.85) |
|  | *P* trend=0.860 | *P* trend<0.001 | *P* trend<0.001 | *P* trend<0.001 |

MVPA, moderate to vigorous physical activity; CVD, cardiovascular disease; HR, hazard ratio; CI, confidence interval.

The multivariable-adjusted model was adjusted for age, sex, race, education level, Townsend deprivation index, smoking status, drinking status, parental history of CVD, self-reported use of antihypertensive drugs, self-reported use of lipid-lowering drugs, self-reported use of insulin and sedentary behavior.

| **Table S22. Independent associations of TyG-WHtR index with incident CVD and all-cause mortality after further adjusting sedentary behavior in multivariable-adjusted model** | | | | |
| --- | --- | --- | --- | --- |
|  | **CVD** | | **All-cause mortality** | |
|  | **Unadjusted HR (95% CI)** | **Adjusted HR (95% CI)** | **Unadjusted HR (95% CI)** | **Adjusted HR (95% CI)** |
| TyG-WHtR (per unit) | 1.48 (1.46-1.49) | 1.24 (1.23-1.26) | 1.38 (1.37-1.40) | 1.14 (1.13-1.16) |
| TyG-WHtR (tertiles) |  |  |  |  |
| Q 1 | 1.00 (Reference) | 1.00 (Reference) | 1.00 (Reference) | 1.00 (Reference) |
| Q 2 | 1.92 (1.85-1.99) | 1.33 (1.28-1.38) | 1.53 (1.47-1.59) | 1.07 (1.02-1.11) |
| Q 3 | 3.17 (3.07-3.27) | 1.69 (1.62-1.76) | 2.31 (2.23-2.39) | 1.27 (1.21-1.32) |
|  | *P* trend<0.001 | *P* trend<0.001 | *P* trend<0.001 | *P* trend<0.001 |

TyG-WHtR, triglyceride glucose-waist height ratio; CVD, cardiovascular disease; HR, hazard ratio; CI, confidence interval.

The multivariable-adjusted model was adjusted for age, sex, race, education level, Townsend deprivation index, smoking status, drinking status, MVPA, parental history of CVD, self-reported use of antihypertensive drugs, self-reported use of lipid-lowering drugs, self-reported use of insulin and sedentary behavior.

| **Table S23. Associations of TyG-WHtR index with incident CVD and all-cause mortality by MVPA after further adjusting sedentary behavior in multivariable-adjusted model** | | | | | | |
| --- | --- | --- | --- | --- | --- | --- |
|  | **CVD** | | | **All-cause mortality** | | |
|  | **Unadjusted**  **HR (95% CI)** | **Adjusted**  **HR (95% CI)** | ***P* for interaction** | **Unadjusted**  **HR (95% CI)** | **Adjusted**  **HR (95% CI)** | ***P* for interaction** |
| <150min/wk |  |  |  |  |  |  |
| TyG-WHtR Q 1 | 1.00 (Reference) | 1.00 (Reference) |  | 1.00 (Reference) | 1.00 (Reference) |  |
| TyG-WHtR Q 2 | 1.91 (1.80-2.03) | 1.35 (1.27-1.43) |  | 1.61 (1.52-1.71) | 1.14 (1.07-1.21) |  |
| TyG-WHtR Q 3 | 3.26 (3.09-3.44) | 1.72 (1.62-1.84) |  | 2.39 (2.26-2.53) | 1.30 (1.21-1.39) |  |
| 150-299min/wk |  |  |  |  |  |  |
| TyG-WHtR Q 1 | 1.00 (Reference) | 1.00 (Reference) | 0.404 | 1.00 (Reference) | 1.00 (Reference) | 0.432 |
| TyG-WHtR Q 2 | 1.93 (1.77-2.10) | 1.31 (1.20-1.44) | 0.427 | 1.40 (1.27-1.53) | 0.95 (0.86-1.05)* | 0.012 |
| TyG-WHtR Q 3 | 3.06 (2.82-3.32) | 1.61 (1.46-1.77) |  | 2.26 (2.07-2.46) | 1.22 (1.09-1.36) |  |
| 300-599min/wk |  |  |  |  |  |  |
| TyG-WHtR Q 1 | 1.00 (Reference) | 1.00 (Reference) | 0.853 | 1.00 (Reference) | 1.00 (Reference) | 0.313 |
| TyG-WHtR Q 2 | 2.00 (1.85-2.17) | 1.36 (1.25-1.48) | 0.853 | 1.58 (1.45-1.72) | 1.08 (0.99-1.19)* | 0.802 |
| TyG-WHtR Q 3 | 3.27 (3.03-3.52) | 1.73 (1.58-1.90) |  | 2.27 (2.09-2.46) | 1.24 (1.12-1.37) |  |
| ≥600min/wk |  |  |  |  |  |  |
| TyG-WHtR Q 1 | 1.00 (Reference) | 1.00 (Reference) | 0.672 | 1.00 (Reference) | 1.00 (Reference) | 0.385 |
| TyG-WHtR Q 2 | 1.85 (1.73-1.98) | 1.27 (1.18-1.37) | 0.783 | 1.44 (1.33-1.55) | 1.00 (0.93-1.09)* | 0.137 |
| TyG-WHtR Q 3 | 2.98 (2.79-3.18) | 1.61 (1.49-1.75) |  | 2.15 (2.00-2.31) | 1.21 (1.11-1.32) |  |

MVPA, moderate to vigorous physical activity; TyG-WHtR, triglyceride glucose-waist height ratio; CVD, cardiovascular disease; HR, hazard ratio; CI, confidence interval.

The P value for interaction was obtained including the product term of TyG-WHtR index (tertiles) and MVPA (four groups) in the multivariable-adjusted model, among which TyG-WHtR Q 3 and <150min/wk MVPA was set as the reference group.

The multivariable-adjusted model was adjusted for age, sex, race, education level, Townsend deprivation index, smoking status, drinking status, parental history of CVD, self-reported use of antihypertensive drugs, self-reported use of lipid-lowering drugs, self-reported use of insulin and sedentary behavior. *Value indicates no statistical significance.

| **Table S24. Joint associations of MVPA and TyG-WHtR with incident CVD and all-cause mortality after further adjusting sedentary behavior in multivariable-adjusted model** | | | | |
| --- | --- | --- | --- | --- |
|  | **CVD** | | **All-cause mortality** | |
|  | **Unadjusted HR (95% CI)** | **Adjusted HR (95% CI)** | **Unadjusted HR (95% CI)** | **Adjusted HR (95% CI)** |
| <150 min/wk MVPA and TyG-WHtR Q 3 | 1.00 (Reference) | 1.00 (Reference) | 1.00 (Reference) | 1.00 (Reference) |
| <150 min/wk MVPA and TyG-WHtR Q 2 | 0.59 (0.56-0.61) | 0.79 (0.75-0.82) | 0.67 (0.64-0.71) | 0.88 (0.84-0.92) |
| <150 min/wk MVPA and TyG-WHtR Q 1 | 0.31 (0.29-0.32) | 0.59 (0.56-0.63) | 0.42 (0.40-0.44) | 0.78 (0.73-0.83) |
| 150-299min/wk MVPA and TyG-WHtR Q 3 | 0.88 (0.84-0.92) | 0.90 (0.86-0.95) | 0.87 (0.82-0.92) | 0.89 (0.84-0.94) |
| 150-299min/wk MVPA and TyG-WHtR Q 2 | 0.55 (0.52-0.59) | 0.73 (0.69-0.78) | 0.54 (0.50-0.57) | 0.69 (0.65-0.74) |
| 150-299min/wk MVPA and TyG-WHtR Q 1 | 0.29 (0.27-0.31) | 0.55 (0.51-0.60) | 0.38 (0.36-0.41) | 0.72 (0.66-0.78) |
| 300-599min/wk MVPA and TyG-WHtR Q 3 | 0.91 (0.87-0.95) | 0.91 (0.87-0.96) | 0.82 (0.77-0.86) | 0.81 (0.76-0.86) |
| 300-599min/wk MVPA and TyG-WHtR Q 2 | 0.56 (0.53-0.59) | 0.72 (0.69-0.76) | 0.57 (0.53-0.60) | 0.72 (0.67-0.76) |
| 300-599min/wk MVPA and TyG-WHtR Q 1 | 0.28 (0.26-0.30) | 0.53 (0.50-0.57) | 0.36 (0.34-0.39) | 0.66 (0.61-0.72) |
| ≥600min/wk MVPA and TyG-WHtR Q 3 | 1.02 (0.97-1.06) | 0.96 (0.92-1.00) | 0.92 (0.87-0.97) | 0.85 (0.80-0.89) |
| ≥600min/wk MVPA and TyG-WHtR Q 2 | 0.63 (0.60-0.66) | 0.75 (0.71-0.79) | 0.61 (0.58-0.65) | 0.70 (0.66-0.74) |
| ≥600min/wk MVPA and TyG-WHtR Q 1 | 0.34 (0.32-0.36) | 0.58 (0.54-0.62) | 0.43 (0.40-0.46) | 0.69 (0.64-0.73) |

MVPA, moderate to vigorous physical activity; TyG-WHtR, triglyceride glucose-waist height ratio; CVD, cardiovascular disease; HR, hazard ratio; CI, confidence interval.

The multivariable-adjusted model was adjusted for age, sex, race, education level, Townsend deprivation index, smoking status, drinking status, parental history of CVD, self-reported use of antihypertensive drugs, self-reported use of lipid-lowering drugs, self-reported use of insulin and sedentary behavior.

| **Table S25. Independent associations of TyG-WC index with incident CVD and all-cause mortality** | | | | |
| --- | --- | --- | --- | --- |
|  | **CVD** | | **All-cause mortality** | |
|  | **Unadjusted HR (95% CI)** | **Adjusted HR (95% CI)** | **Unadjusted HR (95% CI)** | **Adjusted HR (95% CI)** |
| TyG-WC (per SD) | 1.53 (1.52-1.55) | 1.28 (1.27-1.30) | 1.38 (1.37-1.40) | 1.16 (1.15-1.18) |
| TyG-WC (tertiles) |  |  |  |  |
| Q 1 | 1.00 (Reference) | 1.00 (Reference) | 1.00 (Reference) | 1.00 (Reference) |
| Q 2 | 1.86 (1.79-1.92) | 1.31 (1.26-1.36) | 1.43 (1.38-1.48) | 1.02 (0.98-1.06) |
| Q 3 | 2.96 (2.87-3.06) | 1.72 (1.66-1.78) | 2.07 (2.00-2.15) | 1.24 (1.19-1.29) |
|  | *P* trend<0.001 | *P* trend<0.001 | *P* trend<0.001 | *P* trend<0.001 |

TyG-WC, triglyceride glucose-waist circumference; CVD, cardiovascular disease; HR, hazard ratio; CI, confidence interval.

The multivariable-adjusted model was adjusted for age, sex, race, education level, Townsend deprivation index, smoking status, drinking status, MVPA, parental history of CVD, self-reported use of antihypertensive drugs, self-reported use of lipid-lowering drugs and self-reported use of insulin.

The SD of TyG-WC is 145.42.

| **Table S26. Associations of TyG-WC index with incident CVD and all-cause mortality by MVPA** | | | | | | |
| --- | --- | --- | --- | --- | --- | --- |
|  | **CVD** | | | **All-cause mortality** | | |
|  | **Unadjusted**  **HR (95% CI)** | **Adjusted**  **HR (95% CI)** | ***P* for interaction** | **Unadjusted**  **HR (95% CI)** | **Adjusted**  **HR (95% CI)** | ***P* for interaction** |
| <150min/wk |  |  |  |  |  |  |
| TyG-WC Q 1 | 1.00 (Reference) | 1.00 (Reference) |  | 1.00 (Reference) | 1.00 (Reference) |  |
| TyG-WC Q 2 | 1.91 (1.80-2.03) | 1.36 (1.28-1.44) |  | 1.46 (1.37-1.55) | 1.05 (0.98-1.12)* |  |
| TyG-WC Q 3 | 3.03 (2.87-3.21) | 1.72 (1.62-1.83) |  | 2.07 (1.96-2.19) | 1.23 (1.16-1.31) |  |
| 150-299min/wk |  |  |  |  |  |  |
| TyG-WC Q 1 | 1.00 (Reference) | 1.00 (Reference) | 0.140 | 1.00 (Reference) | 1.00 (Reference) | 0.910 |
| TyG-WC Q 2 | 1.77 (1.63-1.93) | 1.22 (1.12-1.34) | 0.712 | 1.32 (1.20-1.44) | 0.92 (0.83-1.01)* | 0.011 |
| TyG-WC Q 3 | 2.80 (2.59-3.04) | 1.58 (1.45-1.73) |  | 2.06 (1.90-2.25) | 1.20 (1.09-1.33) |  |
| 300-599min/wk |  |  |  |  |  |  |
| TyG-WC Q 1 | 1.00 (Reference) | 1.00 (Reference) | 0.390 | 1.00 (Reference) | 1.00 (Reference) | 0.538 |
| TyG-WC Q 2 | 1.91 (1.77-2.07) | 1.33 (1.23-1.44) | 0.095 | 1.46 (1.35-1.59) | 1.02 (0.93-1.11)* | 0.818 |
| TyG-WC Q 3 | 3.18 (2.95-3.42) | 1.84 (1.69-2.00) |  | 2.04 (1.88-2.21) | 1.20 (1.09-1.31) |  |
| ≥600min/wk |  |  |  |  |  |  |
| TyG-WC Q 1 | 1.00 (Reference) | 1.00 (Reference) | 0.641 | 1.00 (Reference) | 1.00 (Reference) | 0.644 |
| TyG-WC Q 2 | 1.80 (1.69-1.93) | 1.26 (1.18-1.35) | 0.281 | 1.41 (1.31-1.51) | 1.01 (0.93-1.09)* | 0.227 |
| TyG-WC Q 3 | 2.83 (2.66-3.02) | 1.67 (1.55-1.79) |  | 2.01 (1.87-2.16) | 1.25 (1.15-1.35) |  |

MVPA, moderate to vigorous physical activity; TyG-WC, triglyceride glucose-waist circumference; CVD, cardiovascular disease; HR, hazard ratio; CI, confidence interval.

The P value for interaction was obtained including the product term of TyG-WC index (tertiles) and MVPA (four groups) in the multivariable-adjusted model, among which TyG-WC Q 3 and <150min/wk MVPA was set as the reference group.

The multivariable-adjusted model was adjusted for age, sex, race, education level, Townsend deprivation index, smoking status, drinking status, parental history of CVD, self-reported use of antihypertensive drugs, self-reported use of lipid-lowering drugs and self-reported use of insulin. *Value indicates no statistical significance.

| **Table S27. Joint associations of MVPA and TyG-WC with incident CVD and all-cause mortality** | | | | |
| --- | --- | --- | --- | --- |
|  | **CVD** | | **All-cause mortality** | |
|  | **Unadjusted HR (95% CI)** | **Adjusted HR (95% CI)** | **Unadjusted HR (95% CI)** | **Adjusted HR (95% CI)** |
| <150 min/wk MVPA and TyG-WC Q 3 | 1.00 (Reference) | 1.00 (Reference) | 1.00 (Reference) | 1.00 (Reference) |
| <150 min/wk MVPA and TyG-WC Q 2 | 0.63 (0.60-0.66) | 0.78 (0.75-0.82) | 0.71 (0.67-0.74) | 0.85 (0.81-0.89) |
| <150 min/wk MVPA and TyG-WC Q 1 | 0.33 (0.31-0.35) | 0.58 (0.55-0.62) | 0.48 (0.46-0.51) | 0.82 (0.77-0.87) |
| 150-299min/wk MVPA and TyG-WC Q 3 | 0.91 (0.86-0.95) | 0.91 (0.87-0.96) | 0.89 (0.84-0.94) | 0.89 (0.84-0.95) |
| 150-299min/wk MVPA and TyG-WC Q 2 | 0.57 (0.54-0.61) | 0.70 (0.67-0.75) | 0.57 (0.53-0.61) | 0.67 (0.63-0.72) |
| 150-299min/wk MVPA and TyG-WC Q 1 | 0.32 (0.30-0.35) | 0.57 (0.53-0.61) | 0.43 (0.40-0.46) | 0.73 (0.67-0.78) |
| 300-599min/wk MVPA and TyG-WC Q 3 | 0.96 (0.92-1.01) | 0.95 (0.90-0.99) | 0.83 (0.79-0.88) | 0.81 (0.76-0.86) |
| 300-599min/wk MVPA and TyG-WC Q 2 | 0.58 (0.55-0.61) | 0.70 (0.66-0.73) | 0.60 (0.56-0.64) | 0.70 (0.65-0.74) |
| 300-599min/wk MVPA and TyG-WC Q 1 | 0.30 (0.28-0.32) | 0.53 (0.49-0.56) | 0.41 (0.38-0.44) | 0.68 (0.64-0.73) |
| ≥600min/wk MVPA and TyG-WC Q 3 | 1.07 (1.02-1.12) | 0.98 (0.94-1.02) | 0.95 (0.90-1.01) | 0.86 (0.81-0.90) |
| ≥600min/wk MVPA and TyG-WC Q 2 | 0.68 (0.65-0.72) | 0.74 (0.71-0.78) | 0.67 (0.63-0.71) | 0.69 (0.65-0.73) |
| ≥600min/wk MVPA and TyG-WC Q 1 | 0.38 (0.36-0.40) | 0.58 (0.55-0.62) | 0.47 (0.45-0.51) | 0.68 (0.64-0.73) |

MVPA, moderate to vigorous physical activity; TyG-WC, triglyceride glucose-waist circumference; CVD, cardiovascular disease; HR, hazard ratio; CI, confidence interval.

The multivariable-adjusted model was adjusted for age, sex, race, education level, Townsend deprivation index, smoking status, drinking status, parental history of CVD, self-reported use of antihypertensive drugs, self-reported use of lipid-lowering drugs and self-reported use of insulin.


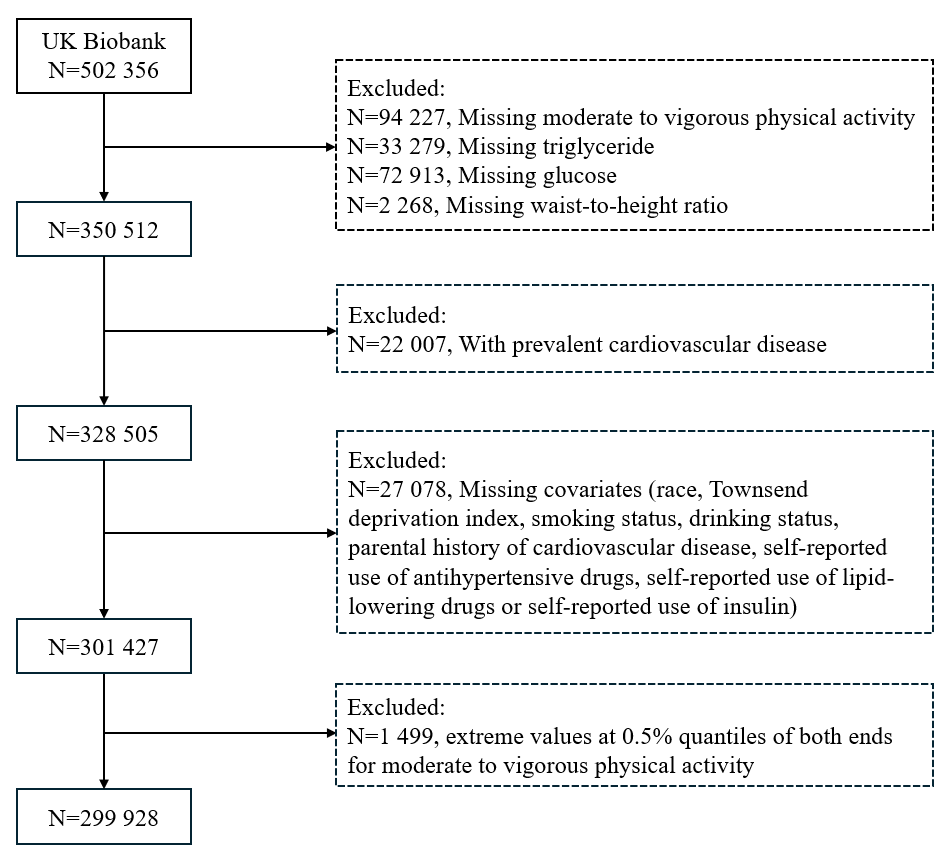


**Fig.** **S1. Flowchart of participant screening**

**
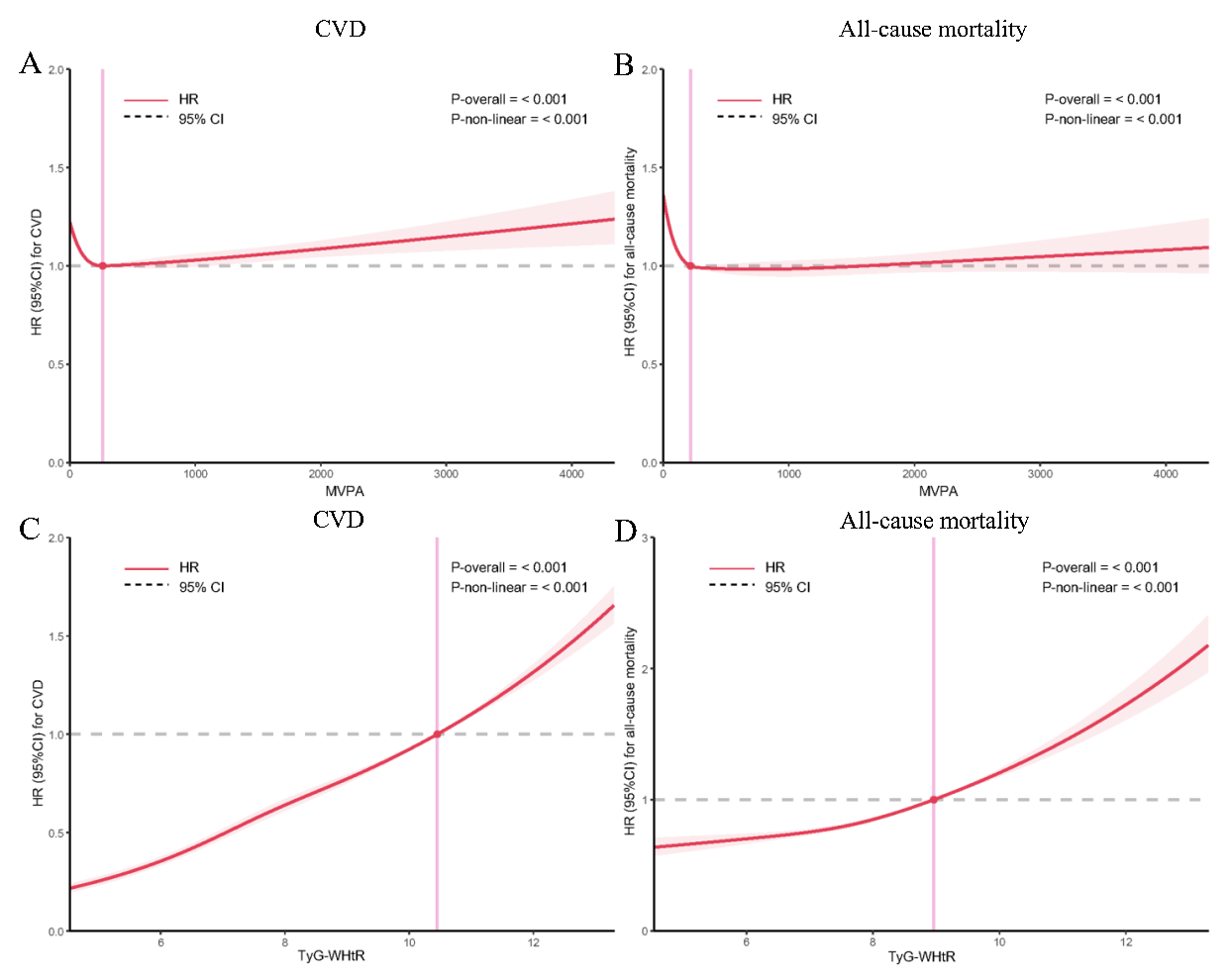
**

**Fig. S2.** **Dose-response associations of MVPA (A, B) and TyG-WHtR index (C, D) with incident CVD and all-cause mortality**

MVPA, moderate to vigorous physical activity; TyG-WHtR, triglyceride glucose-waist height ratio; CVD, cardiovascular disease; HR, hazard ratio; CI, confidence interval. The multivariable-adjusted model was adjusted for age, sex, race, education level, Townsend deprivation index, smoking status, drinking status, parental history of CVD, self-reported use of antihypertensive drugs, self-reported use of lipid-lowering drugs and self-reported use of insulin.


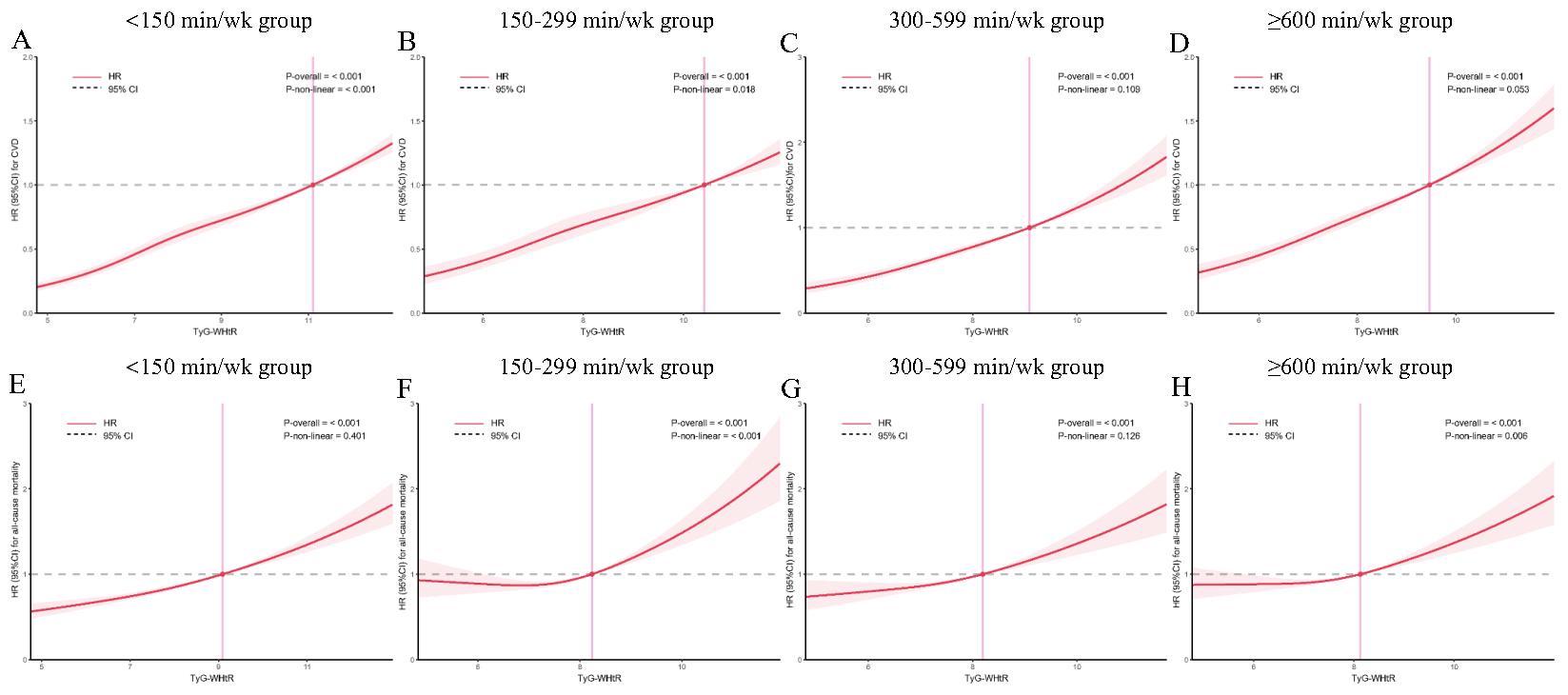


**Fig.** **S3. Dose-response associations of TyG-WHtR index with incident CVD (A-D) and all-cause mortality (E-H) stratified by MVPA**

MVPA, moderate to vigorous physical activity; TyG-WHtR, triglyceride glucose-waist height ratio; CVD, cardiovascular disease; HR, hazard ratio; CI, confidence interval. The multivariable-adjusted model was adjusted for age, sex, race, education level, Townsend deprivation index, smoking status, drinking status, parental history of CVD, self-reported use of antihypertensive drugs, self-reported use of lipid-lowering drugs and self-reported use of insulin.
